# Of Men and Mice: Modeling the Fragile X Syndrome

**DOI:** 10.3389/fnmol.2018.00041

**Published:** 2018-03-15

**Authors:** Regina Dahlhaus

**Affiliations:** Institute for Biochemistry, Emil-Fischer Centre, University of Erlangen-Nürnberg, Erlangen, Germany

**Keywords:** Fragile X Syndrome, mouse model, FMR1, microsatellite instability, E/I balance, behavior and cognition, primates, autism spectrum disorders

## Abstract

The Fragile X Syndrome (FXS) is one of the most common forms of inherited intellectual disability in all human societies. Caused by the transcriptional silencing of a single gene, the fragile x mental retardation gene *FMR1*, FXS is characterized by a variety of symptoms, which range from mental disabilities to autism and epilepsy. More than 20 years ago, a first animal model was described, the *Fmr1* knock-out mouse. Several other models have been developed since then, including conditional knock-out mice, knock-out rats, a zebrafish and a drosophila model. Using these model systems, various targets for potential pharmaceutical treatments have been identified and many treatments have been shown to be efficient in preclinical studies. However, all attempts to turn these findings into a therapy for patients have failed thus far. In this review, I will discuss underlying difficulties and address potential alternatives for our future research.

## Introduction

The tremendous advance that has taken place in life sciences during the last decades has opened a variety of options and opportunities for research as well as for human societies, in particular in the field of genetics. One of these advances was the invention of the Crispr-Cas system (Crispr; reviewed in Donohoue et al., 2017; Huang et al., 2017; Petersen, 2017). The technique allows for a fast and relatively precise gene editing in a variety of different organisms ranging from plants and insects to vertebrates and primates including human cell lines and embryos. Being relatively efficient and easy to use, the system promises much progress not only for our understanding of complex biological systems, but also for the treatment of genetic disorders.

Of foremost interest in this context are monogenetic diseases with limited treatment options for patients, such as the Fragile X Syndrome (FXS), a nonetheless strikingly complex autism spectrum disorder (ASD). Combined with the advance of genetic screening methods for persons at risk (reviewed in Rajan-Babu and Chong, 2016) and assisted reproduction services, the Crispr technique opens not only new horizons, but also raises many ethical concerns, although there is no common agreement on ethical standards among mankind and not all people are sharing the concerns.

For our future research, it will therefore be important to critically evaluate what we are able to achieve, what we have achieved, and, on a society based level, what we do want to achieve. This article will review and discuss important results as well as ideas from the FXS field, and in particular address underlying difficulties arising from the current mouse models.

## The Fragile X Syndrome — of Men

### Phenotype

Affecting approximately 1 in 7000 males and 11,000 females (meta-analysis: Hunter et al., 2014), FXS represents one of the most frequent forms of monogenetically determined mental retardation in all human populations and ethnic groups (reviewed in Tzschach and Ropers, 2007). In the vast majority of cases, the disease is caused by the transcriptional silencing of a single gene on the X chromosome, the Fragile X Mental Retardation gene *FMR1*. In consequence, expression of the encoded protein FMRP is lost (reviewed in Saldarriaga et al., 2014; Usdin and Kumari, 2015).

**Figure 1:**
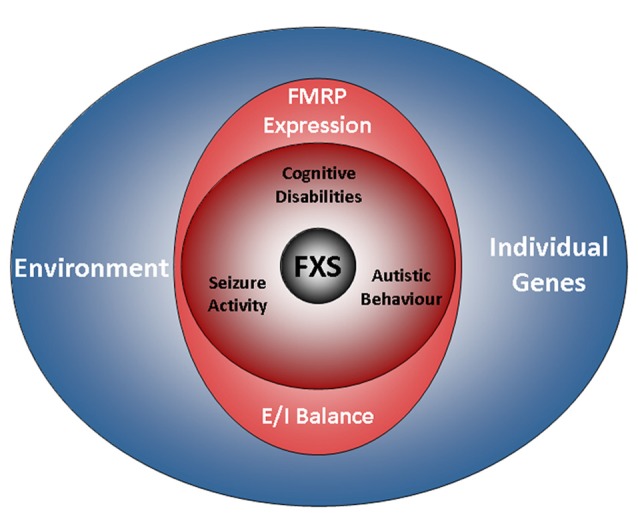
The FXS world. The drawing illustrates factors influencing the disease. A characteristic of FXS is the presence of a broad range of deficits with a high degree of individual variation. The phenotype of the disorder includes cognitive disabilities as well as autistic behaviors and epilepsy. Aside from a loss of FMRP expression, the genetic background and environmental factors are emerging as determinants of the disease. However, while residual FMRP expression is known to correlate with the cognitive performance of FXS patients, the impact of individual genes and the relevance of the environment are less well understood (also see the section “Phenotype”). Recent findings indicate that autistic behaviors and epilepsy are influenced by the E/I balance, but not by residual FMRP expression. FXS, Fragile X Syndrome; FMRP, Fragile X Mental Retardation Protein; E/I balance, balance of excitation and inhibition in neuronal networks.

FXS patients display a variety of intellectual deficits ranging from mild learning impairments to severe cognitive disabilities, but also autistic behaviors such as aggression, social anxiety and stereotypic acting characterize the disease (reviewed in Saldarriaga et al., 2014; Gross et al., 2015b; Figure 1). Men are in general more severely affected than women, achieving only average IQs^1^ of 40–50, whereas women mainly present with mild to moderate cognitive impairments and an average IQ of about 80, though their abilities may range from severe deficits to superior performances (Freund et al., 1993; de Vries et al., 1996; Lewis et al., 2006; Chaste et al., 2012, reviewed in Huddleston et al., 2014). However, even men can be high functioning (Basuta et al., 2015).

Shyness, poor eye contact and attention difficulties are particularly characteristic to young women with FXS (*FMR1^−/–^*), whereas increased aggression and the use of rote phrases are more typical to men (Murphy and Abbeduto, 2007; Hartley et al., 2011). In their adult life, women are mostly affected by deficits in interpersonal skills, while weak functional skills primarily concern men, though reduced social interaction skills are also prominent in men (Hartley et al., 2011). As a result, only 9% of the men affected by FXS achieve a high or very high level of independence in adult life, whereas 44% of the FXS women reach such a level. Contrary to most other X-linked diseases, approximately 35% of the women carrying a single mutated allele only (*FMR1*^–/+^) also demonstrate cognitive disabilities (Hagerman et al., 1992).

The impairments observed in FXS patients are not uniform though, but rather specific to certain capabilities: several studies demonstrated that FXS patients perform particularly weak in tasks requiring abstract item reasoning, attention, the solution of new problems and goal-directed actions, as well as in tasks relying on short-term memory and visual-motor coordination. By contrast, FXS patients usually demonstrate normal skills in vocabulary knowledge, although they show a cluttered and less complex speech (Hanson et al., 1986; Dykens et al., 1987; Maes et al., 1994; Loesch et al., 2004; Lewis et al., 2006; Roberts et al., 2007; Van der Molen et al., 2010).

Recent research has linked some of the variability observed in the cognitive phenotype of FXS patients to residual FMRP expression and mosaic expression patterns (Kaufmann et al., 1999; Dyer-Friedman et al., 2002; Loesch et al., 2003, 2004; Pretto et al., 2014; Basuta et al., 2015). For instance, the cognitive abilities of FXS patients were shown to strongly correlate with FMRP expression levels, even when full-scale IQ scores are used. Although this holds true for most of the cases, exceptions exist: Govaerts and colleagues reported a case, in which residual FMRP expression could not explain the good cognitive performance observed (Govaerts et al., 2007), thus implying a role for individual genetic factors and/or environmental effects (cp.^2^ Figure 1).

Further studies indeed support the significance of environmental factors in FXS (Dyer-Friedman et al., 2002; Kuo et al., 2002; Glaser et al., 2003): in particular maternal warmth and responsivity were demonstrated to ameliorate maladaptive as well as autistic behaviors, whereas maternal depression and criticism were indicated to increase FXS symptoms in children (Greenberg et al., 2012; Robinson et al., 2016; Smith et al., 2016). Contrary to cognitive deficits, autistic behaviors display no correlation with residual FMRP expression (Glaser et al., 2003; Pretto et al., 2014).

Particularly in children, seizures are frequent as well, affecting about 45% of the adolescent patients between 1 year and 14 years of age (Cowley et al., 2016; Figure 1). After the age of 20, seizure activity decreases, resulting in an overall prevalence of about 24% (Sabaratnam et al., 2001), although there is considerable variation among studies. Interestingly, some data suggest that the attention deficits observed in FXS patients are related to seizure activity (Cowley et al., 2016).

Just like autistic behaviors, seizure activity was found not to correlate with residual FMRP expression (Pretto et al., 2014). Although an imbalance of excitatory and inhibitory neuronal activity has been associated with seizure activity and autistic behaviors in several ASDs as well as in corresponding animal models (reviewed in Frye et al., 2016; Uzunova et al., 2016; Lee et al., 2017), data on FXS patients are rare. Using EEG^3^ electrodes, increases in event related potentials were found in the auditory cortex of FXS children, suggesting enhanced excitability (Castrén et al., 2003). EEG-studies of oscillatory dynamics in males with FXS identified impaired theta oscillations indicative of an imbalance in excitatory and inhibitory neuronal circuit activity (Van der Molen and Van der Molen, 2013; Van der Molen et al., 2014) and implied a lack of coordination in information processing. Notably, two studies also found decreased activations, one in prefrontal regions and one in the fusiform gyrus (Dalton et al., 2008; Holsen et al., 2008). These findings suggest that FXS patients experience brain-region and most likely circuit-specific imbalances in neuronal excitation.

### Genotype

#### Microsatellites — Sources of Complexity

**Figure 2:**
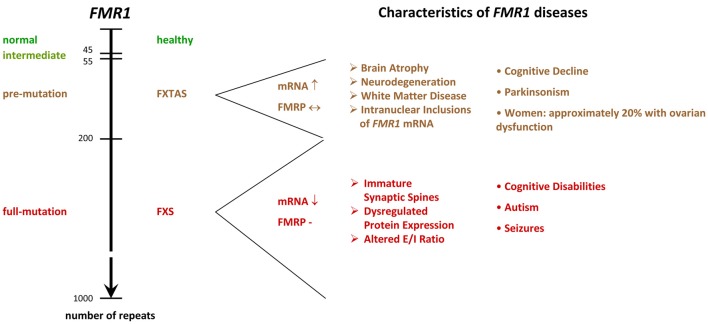
*FMR1*^−/y^ diseases. The scheme shows the relation between microsatellite length (number of repeats) and phenotype. While healthy individuals show 6–44 tandem tracts in the 5′ UTR of their *FMR1* gene, FXS patients display more than 200 repeats. Alleles containing 45–54 repeats are classified as intermediate, and 55–200 repeats as pre-mutation alleles. Premutation alleles give rise to a neurodegenerative disorder called FXTAS, which presents with parkinsonism and brain atrophy. FXTAS typically manifests in individuals over the age of 50. *FMR1*: Fragile X Mental Retardation gene. FMRP: Fragile X Mental Retardation Protein. FXS: Fragile X Syndrome. FXTAS: Fragile X-associated Tremor/Ataxia Syndrome. E/I balance: balance of excitation and inhibition in neuronal networks, UTR: untranslated region. ↓: decreased levels in the diseased condition. ↔ similar levels in normal and diseased conditions. ↑ increased levels in diseased conditions.

The molecular mechanisms leading to the silencing of the *FMR1* gene during embryonic development are complex and result from expansions in the length of a microsatellite located in the 5′UTR^4^ of *FMR1* (Fu et al., 1991; Pieretti et al., 1991; Verkerk et al., 1991; Eiges et al., 2007; Bar-Nur et al., 2012). In healthy individuals, the sequence consists of CGG/CCG tandem tracts and includes approximately 6–44 repeats, whereas FXS patients show more than 200 repeats. *FMR1* alleles containing 45–54 repeats are classified as intermediate, and 55–200 repeats as pre-mutation alleles (Figure 2). Contrary to regular tandem tracts, pre-mutation alleles are meiotically as well as mitotically unstable and may turn into full-mutation alleles within one generation, if transmitted by a female (Fu et al., 1991; Heitz et al., 1992; Yu et al., 1992).

Although microsatellites are often associated with diseases (reviewed in Nelson et al., 2013; Zhang and Ashizawa, 2017), they turned out to play crucial roles in many species, but in particular in humans. Due to their high variability, tandem tracts are thought to serve as a substrate for evolution (reviewed in Kashi and King, 2006; Hannan, 2012; Plohl et al., 2012). Most microsatellites are nonetheless only maintained by chance and total microsatellite numbers are rather species or clade specific than related to vertebrate evolution (Buschiazzo and Gemmell, 2010; Adams et al., 2016). Yet, tract length polymorphism turned out to be a major source for the emergence of variability and complexity in species: recent research revealed that tandem tracts located in regulatory regions contribute to the genesis of complexity (Liu H. et al., 2012; Namdar-Aligoodarzi et al., 2015). By triggering the formation of secondary structures in DNA as well as RNA molecules in a length dependent manner, these repeats redress the transcription and translation efficiency and, in doing so, control the expression level of their proteins (reviewed in Kashi and King, 2006; Pezer et al., 2012; Sawaya et al., 2012; also see e.g., Zumwalt et al., 2007; Halder et al., 2009; Vinces et al., 2009; Quilez et al., 2016). In consequence, the protein stoichiometry is altered, leading to modifications in a variety of protein interactions, while the functionality of the protein itself is preserved. It is therefore no surprise that tandem tracts are preferentially located in the proximity of transcription starts (Vinces et al., 2009; Sawaya et al., 2013; Liang K. C. et al., 2015), that human promoter regions are particularly rich in microsatellites (Sawaya et al., 2013) and that the polymorphism and complexity of tandem tracts significantly increase in primates and humans (Zhang et al., 2004; Mohammadparast et al., 2014; Sabino et al., 2014; Bilgin Sonay et al., 2015; Namdar-Aligoodarzi et al., 2015; Ohadi et al., 2015; Rezazadeh et al., 2015).

Moreover, tandem tract polymorphism turned out to be astonishingly abundant in genes involved in the development of the nervous system (Riley and Krieger, 2009). Indeed, some studies even associated repeat variations with individual differences in behavioral traits—not only in humans (Gerra et al., 2005; Larsen et al., 2010; Simmons and Roney, 2011; Berry et al., 2013; Durdiaková et al., 2013; Valomon et al., 2014; Votinov et al., 2015), but also in birds (Stuber et al., 2016) and other mammals (Hammock and Young, 2005; Lucarelli et al., 2017). In addition, Bagshaw and colleagues showed that polymorphic microsatellites of genes involved in human personality traits and social behavior are able to integrate interactions with the environment, in this case maternal smoking, which caused anti-social acts in carriers of certain TBR1 alleles (Bagshaw et al., 2017). Their finding is in line with an earlier study showing that a CGG repeat variant of the glutathion peroxidase 1 gene is protective for autism (Ming et al., 2010). Indeed, it has been found that prenatal oxidative stress, such as caused by environmental toxicants, is involved in the establishment of autism, in particular when occurring in certain sensitive stages of embryogenesis (reviewed in Chauhan and Chauhan, 2006; Landrigan, 2010; Wells et al., 2016; Heyer and Meredith, 2017).

These findings illustrate that microsatellite polymorphism is an important component of individuality, complexity, neuronal development and gene-environment interactions. Since FMRP itself is developmentally regulated (Hinds et al., 1993) and was found to function in neuronal migration, differentiation and dendritic spine maturation (Hinton et al., 1991; Irwin et al., 2001; Saffary and Xie, 2011; Telias et al., 2013; La Fata et al., 2014; Khalfallah et al., 2017), it seems therefore possible that the microsatellite of *FMR1* might have currently unrecognized functions in the individual peculiarities characteristic to FXS.

#### To Silence or Not to Silence Expanded FMR1?

##### Transcript toxicity in humans

Mirroring the results on microsatellites and protein expression, the different *FMR1* alleles indeed give rise to different expression patterns: pre-mutation carriers are characterized by enhanced mRNA, but normal or slightly reduced protein levels, since the elongated transcripts are inefficiently translated, but heavily transcribed (Tassone et al., 2000a,b; Kenneson et al., 2001; Primerano et al., 2002; Ludwig et al., 2014), whereas full-mutations cause FMRP deficiency due to DNA hypermethylation, Histone modification and subsequent heterochromatin formation (Pieretti et al., 1991; Sutcliffe et al., 1992; Hornstra et al., 1993; Coffee et al., 1999, 2002; Kumari and Usdin, 2010). Some residual mRNA is nonetheless still present in many men with FXS, but the mRNA is not translated (Tassone et al., 2001), probably due to secondary structure formation in the tandem tract.

It is noteworthy that pre-mutation carriers often develop a neurodegenerative disorder called the Fragile X-associated Tremor/Ataxia Syndrome (FXTAS, Figure 2), which presents with neurodegeneration, parkinsonism and brain atrophy, and which is associated with primary ovarian insufficiency in females (reviewed in Botta-Orfila et al., 2016; Hagerman and Hagerman, 2016). FXTAS is believed to arise from a toxicity of elongated mRNA transcripts and/or of a cryptic *FMR1* protein derived from CGG repeat triggered non-ATG translation (Handa et al., 2005; Hashem et al., 2009; Chen et al., 2010; Todd et al., 2013). Since the transcript levels are markedly reduced in FXS patients, these findings suggest that the silencing of full-mutation alleles in FXS serves to prevent from toxic effects, however, clear evidence for a toxicity of the full-mutation mRNA or the cryptic protein is missing. Indeed, the identification of several healthy and non-mosaic individuals carrying unmethylated, normally expressing full-mutation alleles (Smeets et al., 1995; Pietrobono et al., 2005; Tabolacci et al., 2008) argues against the idea of mutation-triggered toxicity in humans. Nonetheless, two cases were identified, where expression of a full-mutation gene caused severe FXTAS (Loesch et al., 2012; Santa Maria et al., 2014), thus supporting the idea that a fully mutated *FMR1* transcript can have toxic effects and that elongated transcripts are causative for FXTAS, but not for FXS, although some mRNA is present in many FXS patients (Tassone et al., 2001).

These apparently conflicting cases illustrate that individual genes and/or environmental effects may overcome the typical mechanisms and phenotypes observed in FXS. The relevance of the latter is further emphasized by the fact that alcohol abuse seemed to be involved in the case of severe FXTAS reported by Loesch and colleagues (Loesch et al., 2012). Recent studies revealed that alcohol is in fact exaggerating behavioral problems such as aggression and impulsivity in FXS patients (Salcedo-Arellano et al., 2016) and accelerating neurological deterioration in FXTAS (Muzar et al., 2014). It might be for these negative effects that, in contrast to cases of high-functioning autism, where patients used drinking to cope with social anxiety (Lalanne et al., 2015), alcoholism is at least in FXS patients rare (cp. Salcedo-Arellano et al., 2016).

For our future research, it will therefore be important to address questions such as: Why may fully mutated *FMR1* transcripts have toxic effects in one case, but not in the other? How does the environment influence the underlying mechanisms? Why may it be better to have a fully mutated gene silenced, and thus FXS, than unsilenced, and eventually FXTAS — from an evolutionary point of view? A deeper understanding of the relevance of genetic individuality is required before attempting a reactivation of *FMR1* in patients (reviewed in Tabolacci et al., 2016b) — in particular, since full-mutation mRNA is not necessarily translated (Tassone et al., 2001; Dolskiy et al., 2017) and since transcribed alleles are able to cause FXTAS under circumstances currently unknown (Loesch et al., 2012; Santa Maria et al., 2014).

##### A question of secondary structures?

An alternative explanation for the silencing of full-mutation alleles is DNA stability. During germ cell generation as well as during early phases of embryogenesis and prior to the silencing of *FMR1*, CGG repeats may expand or contract through mechanisms under debate (reviewed in Mor-Shaked and Eiges, 2016; Gerhardt, 2017). Despite some uncertainty about the exact molecular events that cause repeat instability, it is believed that the formation of secondary DNA structures during recombination, DNA replication and DNA repair leads to the addition or deletion of repeats. Since no repeat instability has been observed in *FMR1* postnatally (Reyniers et al., 1993, 1999; Wöhrle et al., 1993), it is thought that the instability is related to events of the embryogenesis.

Little is known about the factors that could contribute to the instability of the CGG repeats in *FMR1*. It has been noticed that the number of repeats, the content of interspersed AGG and the haplotype are able to influence the stability of *FMR1* (Oberlé et al., 1991; Eichler et al., 1994; Gunter et al., 1998; Hirst and White, 1998; Taylor et al., 1999; Larsen et al., 2000; Dombrowski et al., 2002; Nolin et al., 2003, 2013, 2015; Yrigollen et al., 2012, 2013, 2014; Avitzour et al., 2014; Weiss et al., 2014). AGG interruptions, for instance, have been indicated to support the stability of *FMR1* by reducing secondary structure formation (Weisman-Shomer et al., 2000; Jarem et al., 2010) and promoting appropriate DNA conformations (Jarem et al., 2010) as well as adequate DNA packing (Mulvihill et al., 2005; Volle and Delaney, 2013). Furthermore, the number of repeats was shown to directly correlate with the instability of the tandem tracts (Oberlé et al., 1991; Eichler et al., 1994; Taylor et al., 1999; Avitzour et al., 2014): it is assumed that the size of G-rich tracts directly correlates with the formation of secondary structures and polymerase slippage during replication (Mornet et al., 1996; Freudenreich et al., 1997; Weitzmann et al., 1997; Hirst and White, 1998). In line with this idea, the total length of the CGG repeat allele turned out to be the best predictor for the risk of transmission (Yrigollen et al., 2014).

Aside from internal genetic properties, trans-acting factors have also been postulated to affect the stability of *FMR1* during mitosis and meiosis (Mornet et al., 1996; Nolin et al., 1996, 1999), but although it seems plausible that other genes might impact on the stability of *FMR1*, studies are rare. In an attempt to identify such trans-acting factors, Xu et al. (2013) analyzed two microarray sets containing data on the transcript expression in FXS patients and controls, and found a significant down-regulation of DNA damage/repair pathway transcripts, thus implying that impaired DNA repair pathways may support *FMR1* instability in FXS patients.

Remarkably, environmental factors have also been found to influence the instability of *FMR1*: maternal age was recently related to increased instability (Yrigollen et al., 2014) and oxidative stress was demonstrated to interfere with the stability of *FMR1* (Adihe Lokanga et al., 2014). The latter finding is in line with other studies showing that different kinds of stress can induce instability in microsatellites (Chatterjee et al., 2015; Wu et al., 2017) and that oxidized DNA can trigger repeat expansion or contraction (Lai et al., 2013; Cilli et al., 2016). Given that chronic alcoholism causes oxidative stress (reviewed in Wu et al., 2006; Hernández et al., 2016), the data imply that alcohol abuse could contribute to the consolidation of *FMR1* diseases. Interestingly, a study conducted by Kogan and colleagues found alcoholism to be significantly more common in families of pre-mutation carriers than in control families (Kogan et al., 2008). Although this association does not tell whether the disease (FXTAS) is causing the alcoholism, for example by overcharging family members who care for their affected relatives, or whether the alcoholism is causing the disease (or both), the facts that maternal alcoholism seems to increase the risk for FXS (mentioned in Hagerman et al., 2010) and that foetuses are particularly vulnerable to alcohol (reviewed in Henderson et al., 1999; Dennery, 2007) support a role for alcohol abuse in the emergence of FXS. Much more research is needed to establish the relation of toxins, such as derived from smoking or drinking, oxidative stress, microsatellite instability and the consolidation of *FMR1* diseases.

Repeat numbers exceeding 200 tandems trigger the epigenetic silencing of *FMR1* by initiating an abnormal 5′-C-phosphate-G-3′ methylation and repressive Histone modifications in the promoter region (e.g., Coffee et al., 1999, 2002; Chandler et al., 2003; Kumari and Usdin, 2010; Brasa et al., 2016). As a consequence, CpG^5^ islands flanking the repeats as well as the repeats itself, which also function as a CpG island, become hypermethylated and render the gene inactive (Hansen et al., 1992). Studies in human FXS cell lines aiming to reactivate *FMR1* by either changing repressive Histone modifications (Kumari and Usdin, 2016; Dolskiy et al., 2017) or decreasing CpG methylation (Chiurazzi et al., 1998; Coffee et al., 2002; Tabolacci et al., 2005, 2016a), suggest that DNA methylation is the primary cause for gene inactivity, while repressive Histone methylations have a supportive function.

The molecular mechanisms by which CGG expansions trigger the epigenetic silencing of *FMR1* are currently not well understood though (for a review, please see Usdin and Kumari, 2015). It is thought that transient unpairing of the DNA during replication, transcription or repair provides an opportunity for the repeat region of *FMR1* to form secondary structures such as hairpins or G-quadruplexes (Fry and Loeb, 1994; Kettani et al., 1995; Mitas et al., 1995; Usdin and Woodford, 1995; Patel et al., 2000; Loomis et al., 2014). Similar structures occur in *FMR1* transcripts (Handa et al., 2003; Napierala et al., 2005; Zumwalt et al., 2007; Malgowska et al., 2014). Studies showed that these secondary structures hinder replication, transcription and translation (Fry and Loeb, 1994; Nadel et al., 1995; Usdin and Woodford, 1995; Subramanian et al., 1996) and that structure-disrupting proteins are able to alleviate the situation (Fukuda et al., 2005; Khateb et al., 2007), thus suggesting that the formation of secondary structures is troubling the cells.

Not all structures are alike though: R-loops, a DNA-RNA hybrid formed during transcription, were recently indicated to prevent gene silencing by protecting DNA from *de novo* methylation (Ginno et al., 2012). Moreover, R-loops were shown to cause chromosome decondensation and transcription activation (Powell et al., 2013). Since *FMR1* has been observed to give rise to R-loops (Groh et al., 2014; Loomis et al., 2014), this data could nicely explain the enhanced expression of pre-mutation alleles observed in FXTAS, but experimental evidence is missing. In fact, Groh and colleagues found that the formation of R-loops on *FMR1* impedes gene expression (Groh et al., 2014), thus suggesting that R-loop formation is involved in the silencing of *FMR1*. Their data are in line with another study demonstrating that promoter-bound *FMR1* transcripts containing tandem tracts induce the silencing of *FMR1* (Colak et al., 2014).

The relevance of the number of repeats required to build R-loops and the role of the respective loop size are not yet clear though, since R-loops were found to form on normal, pre-mutation and full-mutation alleles (Colak et al., 2014; Groh et al., 2014; Loomis et al., 2014). It seems therefore possible that the role of R-loops in transcription regulation depends on the loop size: Loomis and colleagues provided evidence that the expansion of *FMR1* repeats causes an enhanced formation of aggrandized loops, which tend to form higher-order structures. These structures distinguish *FMR1* from other CpG islands containing promoters (Ginno et al., 2012, 2013) and could finally trigger repeat instability and hypermethylation (Loomis et al., 2014). Hence, R-loops formed on *FMR1* could enhance gene expression until they exceed a specific size and form secondary structures. Previous research on the methylation of different loci indeed indicated that it is the higher-order structures that trigger DNA methylation (Smrzka et al., 1995; Paoloni-Giacobino et al., 2007; Gentry and Meyer, 2013).

These findings imply that CpG methylation could serve to limit the formation of secondary structures. Nuclear magnetic resonance analyses revealed CpG methylations to decrease the dynamics of the DNA backbone (Geahigan et al., 2000), while molecular dynamic investigations illustrated DNA methylation to increase the rigidity of the DNA by steric hinderance and hydrophobicity (Derreumaux et al., 2001). Using density functional theory and nuclear magnetic resonance measurements, Taqi and colleagues further demonstrated that cytosine methylation impairs the conformational flexibility of short ssDNA^6^ molecules and their ability to form secondary structures (Taqi et al., 2012). Although these data support the idea that hypermethylation of *FMR1* could serve to prevent the formation of secondary structures, more evidence is required.

Further studies showed that the hypermethylation of *FMR1* indeed correlates with enhanced tract stability during mitosis (Gläser et al., 1999; Wöhrle et al., 2001; Nichol Edamura et al., 2005). Remarkably, Zhou and colleagues observed that cells carrying fully mutated and hypermethylated alleles outcompete those carrying alleles with less repeats and no methylation when co-cultured, resulting in a loss of these cells (Zhou et al., 2016). Since no differences were seen in the viability of both cell lines, toxic effects are unlikely to account for the disappearing of cells with unsilenced alleles. The reasons for this effect remain to be investigated though.

The data imply that hypermethylation of *FMR1* should occur when cells start to divide a lot. Studies using fetal tissues showed that hypermethylation is established between the 10^th^ and 12^th^ week of gestation, but *FMR1* may remain partly active for some time (Devys et al., 1992; Sutcliffe et al., 1992; Suzumori et al., 1993; Iida et al., 1994; Willemsen et al., 2002; reviewed in Mor-Shaked and Eiges, 2018). Models of cell division in human embryos do not support high rates of mitosis during this time though, they rather show a decline in division rates (Luecke et al., 1999). Looking at the brain in specific, the situation is yet different: By the 9^th^ week of gestation, neuronal tube formation is completed and shortly after, at the 12^th^ week of gestation, neurogenesis as well as neuronal migration will reach their first peak (reviewed in Linderkamp et al., 2009). Nerve cells will then be proliferating at rates of about 15 million per hour (reviewed in Ackerman, 1992), thus implying that the silencing of *FMR1* could be related to cell line-specific changes serving in neurogenesis. Such a mechanism could be important to maintain the correct timing and pace during neuronal development, which is essential to establish the complex connections that characterize the brain.

Indeed, when Khalfallah and colleagues induced differentiation in a murine embryonic stem cell line lacking FMRP (sh*Fmr1* ES), they found an accelerated generation of both, progenitor and neuronal cells during the first steps of neurogenesis (Khalfallah et al., 2017; reviewed in Bardoni et al., 2017; Westmark, 2017). Their experiments further revealed that this phenotype is due to enhanced expression of a target of FMRP, APP^7^, which is able to accelerate neurogenesis following cleavage into the A-beta peptide. This mechanism might also provide an alternative or additional explanation for the observation that embryonic stem cells carrying a hypermethylated *FMR1* gene outcompete cells with the active gene as it was found by Zhou and colleagues (Zhou et al., 2016). It will be interesting to see how these changes affect neuronal maturation and signaling and how the findings of Khalfallah and colleagues relate to neuronal development in humans.

Notably, studies employing human embryonic stem cells (hESCs) showed that the epigenetic silencing of *FMR1* may occur prior to differentiation (Avitzour et al., 2014; Zhou et al., 2016) or upon differentiation (Eiges et al., 2007): of 11 hESC lines, 7 showed some levels of stable hypermethylation prior to differentiation. It is not yet clear though, when and how the hypermethylation is established. Current models suggest that fully expanded genes first acquire abnormal methylation patterns before or during embryo implantation and that *FMR1* silencing is achieved after the blastocyst stage. Microsatellites that escape the initial methylation changes are believed to remain unmethylated. Since standard reprogramming procedures serving to generate induced pluripotent stem cells (iPSCs) from skin fibroblasts are unable to remove the exaggerated methylation marks inactivating *FMR1* (Urbach et al., 2010; Sheridan et al., 2011; Doers et al., 2014), hypermethylation is thought to be stable and irreversible once it is established.

This is not necessarily the case though: removing CGG repeats and the immediate 5′-flanking region from fully mutated microsatellites in the *FMR1* gene of a male iPSC line by the CRISPR/Cas9 system, Park and colleagues were able to restore *FMR1* gene expression as well as FMRP protein levels in a pair of clones (Park et al., 2015). The reactivation of *FMR1* led to a stable expression of the gene throughout differentiation into mature neurons. These findings are further supported by the previously mentioned study of Zhou and colleagues, who showed that hypermethylation present in embryonic stem cells is dynamic (Zhou et al., 2016): alleles containing over 400 repeats may contract to smaller repeat numbers, resulting in a permanent reactivation even when more than 200 tandems are present. Moreover, de Esch and colleagues (de Esch et al., 2014) observed that the reprogramming of fibroblasts from an atypical individual carrying an active full mutation *FMR1* gene with 330 repeats into iPSCs recurrently resulted in a complete inactivation of the gene. Taken together, these studies not only illustrate the dynamics of the system, but also suggest that repeat length is not the only factor influencing the silencing of *FMR1* and that other factors, such as neurodevelopmental stage are involved. Indeed, the fact that the brother of the atypical individual also carried an unsilenced full-mutation implies that maternal-parental components, which were not present in fibroblasts or inactivated during the reprogramming procedure, and/or specific environmental factors caused their *FMR1* genes to remain active despite the high repeat length.

Aside from tandem repeat polymorphism, mutations in the coding region of *FMR1* have also been associated with the occurrence of FXS in patients (Quartier et al., 2017): a deletion of the last exon, which is giving rise to a truncated FMRP isoform, was recently identified in three brothers meeting FXS criteria (Hagerman’s scores = 15). Moreover, two splice variants were detected in two unrelated patients showing the same outcome in the test, and several missense mutations have been identified in FXS patients (Siomi et al., 1994; Handt et al., 2014; Myrick et al., 2014). Although these cases represent rare exemptions, they nonetheless demonstrate that the correct functioning of FMRP is central to FXS.

## The Fragile X Syndrome — of Mice

### The Genotype of Mice or the Charm of Simplicity

Mice are much different from humans, they are much smaller, live much shorter and have much less to learn. Nonetheless, mice and men are sharing almost 99% of their genes (Waterston et al., 2002) as well as most physiological functions and pathogenic mechanisms (see for example the reviews of Tecott, 2003; Elefteriou and Yang, 2011; Van der Weyden and Adams, 2013; Jacobs et al., 2013; Eilam, 2014; Hoehndorf et al., 2014; Vandamme, 2014; Lubojemska et al., 2016). Indeed, even their aging was recently shown to match human senescence surprisingly well (Dutta and Sengupta, 2016). Since mice are also easy to keep, they became the most widely used model organism in life sciences after their first documented employment almost 500 years ago (reviewed in Paigen, 2003; Goodman et al., 2015): of nearly 11.5 million animals used for scientific purposes in the European Union in 2011, 61% were mice (European Commission, 2010). Despite these massive research efforts, most attempts to translate the outcomes to humans have failed. In the FXS field for instance, more than 70 studies reporting rescues (excluding reviews) have been published on pubmed during the last 12 years, 63 clinical trials are registered on ClinicalTrials.gov, and not a single treatment is available for patients yet (2^nd^ of October 2017; current state reviewed in Ligsay and Hagerman, 2016). Although several positive outcomes were observed during the trials, indicating at least some progress toward a better understanding of the disease and a treatment for patients, discrepancies between the data obtained in men and mice were common: benefits experienced by patients were often very subtle, limited to subgroups, outside the outcome measures or simply absent. How much wishful-thinking is involved in our mouse models?

Some facts on men and mice (for more details, please see Table 1):

- mice are 3000× smaller
- mice and humans diverted 75 million years ago (Waterston et al., 2002)
- laboratory mice are highly inbred
- their genome is approximately 14% smaller, probably due to deletions (Waterston et al., 2002)
- the DNA sequence identity is only 40% (Waterston et al., 2002)
- the average substitution rate in mice is twofold higher than in humans (Waterston et al., 2002)
- manipulations of their genome may induce mutations in their microsatellite sequences (Zuo et al., 2012; Du et al., 2013)
- mouse-specific promoter and enhancer regions are significantly enriched in repetitive sequences (Yue et al., 2014)
- only 12.6% of the murine DNA are associated with regulatory functions such as transcription factor binding, chromatin organization etc. (humans: 20%; Yue et al., 2014)
- approximately 50% of the regulatory sequences have no identifiable orthologs in human (Cheng et al., 2014; Yue et al., 2014)
- 38.5% of mouse-specific transcription enhancers do not show activity in human ES cells (Yue et al., 2014)
- mice have dozens of local gene family expansions related to reproduction, immunity and olfaction (Waterston et al., 2002)

**Table 1:** Differences between men and mice.

Category	Mice and men	Reference
Transposons	The mouse genome contains only 35.5% of transposon derived DNA (humans: >46%), but with 32.4% an higher amount of lineage-specific repeats (humans: 24.4%).	Waterston et al. (2002)
Breakpoint regions	Evolutionary breakpoint regions (intervals between segments of conserved gene order) of mice are mainly enriched for transposable elements of the SINE type (short interspersed nucleotide elements), whereas human breakpoint regions mainly contain the Alu type, a specific subtype of SINE elements.	Schibler et al. (2006)
Transcription	Only 22% of transcription factor footprints and 50% of transcription factor networks are conserved.	Yue et al. (2014)
	Although the binding motifs of most sequence-specific transcription factors are conserved, the motifs for co-factors tend to be species specific.	Cheng et al. (2014)
Immune system	Differences in the immune system include in the balance of leukocyte subsets, in defensins, Toll receptors, inducible NO synthase, Ig subsets, the B cell and T cell signaling pathways, cytokines and cytokine receptors, Th1/Th2 differentiation, co-stimulatory molecule expression and function, antigen-presenting function of endothelial cells, and chemokine and chemokine receptor expression.	reviewed in Mestas and Hughes (2004)
Physiology	Several differences in the physiology and morphology of organs have been reported recently.	e.g.,: Gharib et al. (2010), Tabata et al. (2012), Barak et al. (2013), Dolenšek et al. (2015), Schmidt et al. (2015) and Symonds et al. (2015)
	Mice are indicated to have higher rates of reactive oxygen species production than humans, however, sufficient original evidence is missing.	Ku et al. (1993), reviewed in Finkel and Holbrook (2000) and Demetrius (2006)
	The fatty acid composition of the membrane is different in mice and men.	Hulbert (2005)

*The table summarizes genetic as well as physiological differences between mice and men, which may potentially affect the translation of research results between the two species. While genome studies in mice and humans showed that both mammals mainly differ in terms of gene regulation, differences in the physiology are not well characterized yet*.

**Table 2:** Fragile X Syndrome and Fragile X-associated Tremor/Ataxia Syndrome model mice.

*Fmr1* mouse model	MGI	Aliases	Strains	First publication	Further publications and information
Fmr1^tm1Cgr^	1857169	Fmr1 KO, Fmr1^tm4Cgr^, FMRP KO, fmr-tm1Cgr, FraX, FMR1-	*FVB.129P-Fmr1^tm1Cgr^/J B6.129P2-Fmr1^tm1Cgr^/J FVB.129P2(B6)-Fmr1^tm1Cgr^/J*	Bakker et al. (1994)	http://www.informatics.jax.org/reference/allele/MGI:1857169?typeFilter=Literature#myDataTable=results%3D100%26startIndex%3D0%26sort%3Dyear%26dir%3Ddesc%26typeFilter%3DLiterature
Fmr1^tm1.1Cidz^	3808885	Fmr1 KO2	B6.129P2- Fmr1^tm1.1Cidz^*/J*	Mientjes et al. (2006)	http://www.informatics.jax.org/reference/allele/MGI:3808885?typeFilter=Literature
Fmr1^tm1Cidz^	3603442	Fmr1 CKO	Involves: 129S1/Sv^*^ 129X1/SvJ	Koekkoek et al. (2005)	http://www.informatics.jax.org/reference/allele/MGI:3603442?typeFilter=Literature
Fmr1^tm2Cgr^	2451086	CGG(98) Fmr1 CGG KI Fmr1 CGG KI (C57BL/6 congenic)	*B6.129P2(Cg)-Fmr1^tm2Cgr^/DlnJ*	Bontekoe et al. (2001)	http://www.informatics.jax.org/reference/allele/MGI:2451086?typeFilter=Literature
Fmr1^tm1Usdn^	3711215	CGG KI, Fmr1^PM^	*B6.129S6(Cg)-Fmr1^tm1Usdn^*	Entezam et al. (2007)	http://www.informatics.jax.org/reference/allele/MGI:3711215?typeFilter=Literature
Fmr1^tm1Rbd^	3840615	Fmr1^I304N^ Fmr1^tm1(I304N)Drnl^ Fmr1^tm1(I304N)Rbd^	FVB.129-Fmr1^tm1Rbd^/J B6.129-Fmr1^tm1Rbd^/J	Zang et al. (2009)	http://www.informatics.jax.org/reference/allele/MGI:3840615?typeFilter=Literature
Tg(Fmr1-EGFP)HP76Gsat	4847053		B6;FVB-Tg(Fmr1-EGFP)HP76Gsat/Mmucd	-	- also see: http://www.informatics.jax.org/reference/allele/MGI:4847053?typeFilter=Literature

*The table shows the mouse models most commonly used in *FMR1* research. Embryonic stem cell lines are not included. The majority of studies (>260) has been performed with the first mouse model, the *Fmr1* KO mouse (Bakker et al., 1994), which is characterized by a complete loss of FMRP and expression of abnormal mRNA (Yan et al., 2004; Mientjes et al., 2006). To investigate a potential effect of the aberrant mRNA, another model, the *Fmr1* KO2 mouse was generated in 2006 (Mientjes et al., 2006), which does not express any mRNA, but no significant differences between the two animal models were found (Gaudissard et al., 2017). By now, the KO2 animals have been employed in more than 20 studies. Along with the *Fmr1* KO2 mouse model, a conditional FXS mouse was developed as well (*Fmr1* CKO), but thus far, the model has only been employed in approximately 10 studies. The knock-in animals have mostly achieved attention as models of FXTAS. In addition, one model of the I304N mutation (Zang et al., 2009), which affects mRNA binding by the KH domain, has been generated. Model mice are available to researchers with generous support from the FRAXA Research Foundation: https://www.fraxa.org/fragile-x-mutant-mouse-facility/*.

**Figure 3:**
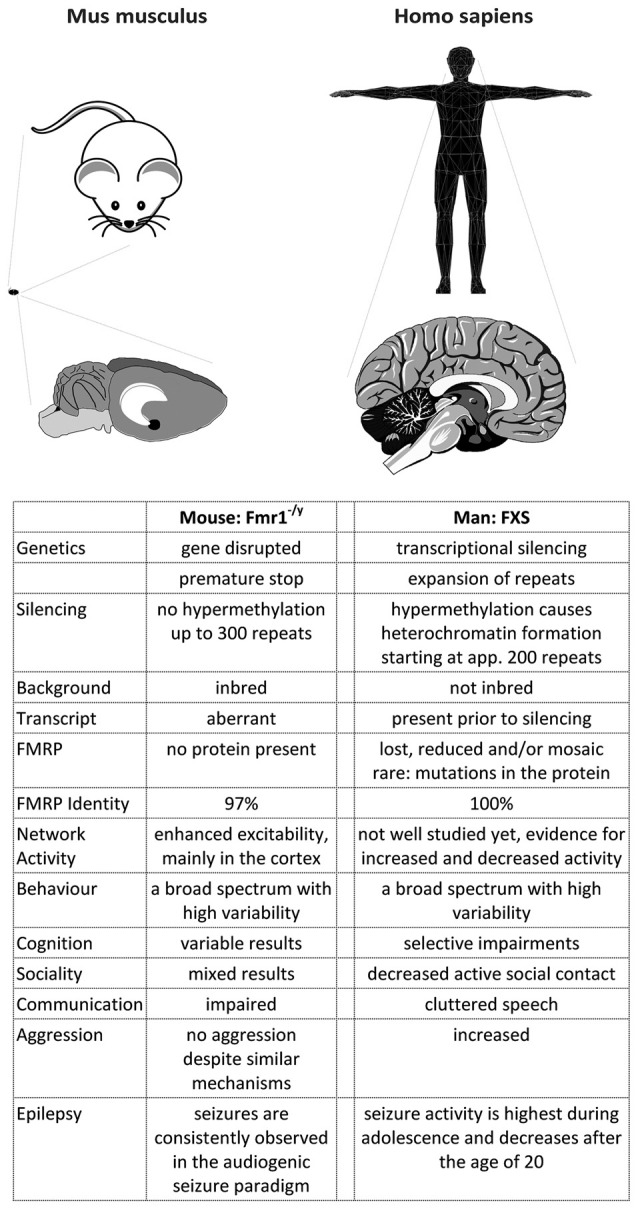
A comparison of FXS in mice and men. The figure summarizes some major differences between FXS model mice and patients. Most differences arise from the development of the cortex in primates, which caused a rewiring inside the cortex as well as between the cortex and the hippocampus (and potentially other brain regions). Consequently, the behavioral phenotype observed in men and mice does not match very well, although the mouse model recapitulates many biochemical aspects of the disease. In addition, the complex genetics of the disease cannot be modeled in mice, probably due to a more relaxed gene expression control in this species.

Taken together, these studies show that although men and mice share many features, they differ in a variety of aspects when more detailed data are included (Figure 3). It is now widely accepted that the characteristics of mice and men mostly arise from alterations in the mechanisms controlling gene expression, in particular from variations and polymorphisms of cis-elements (reviewed in Wittkopp and Kalay, 2011).

### Genetics of the FXS Model Mouse

The fundamental difference between commonly used FXS model mice (*Fmr1*^−/y^ mice^8^) and patients is that mice never have any FMRP, beginning with their very first moment of existence, whereas in patients, the gene is active at least until the 10^th^ week of gestation (Devys et al., 1992; Sutcliffe et al., 1992; Suzumori et al., 1993; Iida et al., 1994; Willemsen et al., 2002). Moreover, patients display mosaic expression patterns (Kaufmann et al., 1999; Dyer-Friedman et al., 2002; Loesch et al., 2003, 2004; Govaerts et al., 2007; Pretto et al., 2014; Basuta et al., 2015) as well as residual full-length mRNA (Tassone et al., 2001), but mice don’t.

In order to overcome this dissatisfying situation, several CGG repeat knock-in mice were made to mimic the genotype found in humans (e.g., Bontekoe et al., 1997, 2001; Lavedan et al., 1997, 1998; Baskaran et al., 2002; Peier and Nelson, 2002; Fleming et al., 2003; Brouwer et al., 2007; Entezam et al., 2007; Alam et al., 2010). Similar to human pre-mutation carriers (Tassone et al., 2000a,b; Kenneson et al., 2001; Primerano et al., 2002; Ludwig et al., 2014), the knock-in mice display a direct correlation between the mRNA transcript level and the repeat length as well as an indirect correlation between the repeat length and the protein level, showing significant variation between individual animals (Ludwig et al., 2014).

In terms of pathological features, the knock-in mice reflect several biochemical, histological and behavioral symptoms of FXTAS patients (Willemsen et al., 2003; Van Dam et al., 2005; Entezam et al., 2007; Hunsaker et al., 2010, 2011; Wenzel et al., 2010; Cunningham et al., 2011) and pre-mutation transcripts were found toxic in mice (Handa et al., 2005; Hashem et al., 2009; Chen et al., 2010; Hukema et al., 2014). However, no explicit data on behavioral deficits or toxic effects were reported for the full-mutation situation (200–350 repeats; Entezam et al., 2007; Hunsaker et al., 2010, 2011; Ludwig et al., 2014) in specific, thus suggesting that contrary to humans, 350 repeats still represent a pre-mutation situation in mice.

Although some instability has been recognized in mice harboring pre-mutation alleles of about 100–260 repeats (Bontekoe et al., 2001; Peier and Nelson, 2002; Brouwer et al., 2007; Entezam et al., 2007), and based on these models, transacting mechanisms such as mismatch repair and transcript coupled repair were identified to support instability of the gene in mice (Zhao and Usdin, 2014; Zhao X. N. et al., 2015), instability is apparently not a major phenotype of the mouse (Bontekoe et al., 1997; Lavedan et al., 1997, 1998; Peier and Nelson, 2002; Fleming et al., 2003).

This situation first changed, when Baskaran and colleagues introduced the SV40 origin of replication along with their transcript to the gene (Baskaran et al., 2002) in order to exclude nucleosome formation. The transgene is driving the expression of *FMR1* Exon 1 by the SV40 early promoter encoded in the SV40 origin, which excludes chromatin formation at the transgenic locus. Exon 1 contains 26 copies of the CGG repeat together with the translational *FMR1* start codon. As a result, the transgene obtained an open chromatin structure compared to the normally nucleosome-flanked CGG repeats seen in other mouse models (Datta et al., 2011) and is therefore more prone to instability (cp. Oostra, 1998). Thereby, Baskaran and colleagues achieved expansions from 26 to 350 repeats within three generations only; however, the DNA remained unmethylated (Alam et al., 2010). Sadly, these results are in line with two more recent studies which also achieved significant expansions (120–230 repeats; Brouwer et al., 2007; Entezam et al., 2007), but no methylation and no inactivation of *Fmr1*. Given that the expression of the full-mutation mRNA did apparently not cause unusual severe FXTAS or even FXS phenotypes, and given that the full-mutation alleles were neither hypermethylated nor inactivated or significantly unstable, these data support the idea that in mice, 200–350 repeats still represent a pre-mutation situation.

Taken together, these studies further suggest that the mechanisms leading to the inactivation of *FMR1* might be different in mice and humans (cp. the section “A Question of Secondary Structures?”). Indeed, Matsuo and colleagues found that CpG islands of men and mice have different properties: comparing 23 orthologous genes, they discovered that mice almost always have less pronounced islands, or even none at all. The authors speculate that the CpG islands in mice might have eroded during evolution as an adaption to the mouse’s small body mass and short life-span by allowing for a more relaxed control of gene activity (Matsuo et al., 1993). Their study is in line with an earlier report that also detected low numbers of CpG islands in mice (Aïssani and Bernardi, 1991), thus implying that mice may simply not have the capabilities to methylate genes the same way humans do, causing fully mutated *Fmr1* to remain active even if humanized transgenes are employed (e.g., Lavedan et al., 1998). This hypothesis is supported by comparative *in vivo* footprinting analyses across several human and mouse CpG islands that demonstrated striking differences in the protein-DNA interactions of both species (Cuadrado et al., 2001, reviewed in Antequera, 2003). Furthermore, Fu and colleagues found that the murine and the human DNA methyltransferase DNMT1, which is responsible for the maintenance of methylation patterns by preferentially adding methyl groups to hemi-methylated CpG sites, differ in their processivity (Fu et al., 2012). In particular, they demonstrate a high level of processivity for human DNMT1 at *FMR1*, which is possibly not achieved by the murine enzyme as it has much longer non-association tracts *in vivo*.

### Phenotypes of the *Fmr1*^−/y^ Mouse

#### FMRP Functions to Regulate the mRNA Metabolism

Studying FMRP functions in humans is challenging. It is therefore no surprise, that most of the corresponding knowledge we acquired is derived from mouse models. However, recent advances in stem cell research contributed significantly to our understanding of the disease.

In healthy individuals, FMRP is widely expressed, yet most abundant in testes and brain, where it is present at particular high levels throughout the cerebral cortex, the hippocampus and the Purkinje cell layer as well as the granular layer of the cerebellum (Devys et al., 1993; Hinds et al., 1993; Bakker et al., 2000, also see http://mouse.brain-map.org/gene/show/14042 and http://www.gensat.org/GeneProgressTracker.jsp?gensatGeneID=339). FMRP has been detected in glial and neuronal cells (Wang et al., 2004; Gholizadeh et al., 2015), but its special importance in synaptic signaling has drawn most attention on its function in neurons. Here, FMRP is primarily located in the cytosol and the nucleus (Feng et al., 1997), but it has also been found along dendrites, axons and at synaptic sites (Weiler et al., 1997; Greenough et al., 2001; Antar et al., 2006; Akins et al., 2017). Indeed, FMRP travels between these locations through microtubules (Feng et al., 1997; Antar et al., 2005).

The main function of FMRP is to regulate the mRNA metabolism. Interacting with five different RNA-motifs (U-Pentameres, Kissing complex, SosLip, G-quartets, G-rich regions), the protein may associate with a diversity of mRNAs, approximately 4% of all mRNAs in the mammalian brain (Brown et al., 2001; Miyashiro et al., 2003; Darnell et al., 2011). Detailed biochemical studies revealed that thereby, FMRP is able to regulate not only mRNA transport (Dictenberg et al., 2008; Estes et al., 2008) and stability (Bagni and Greenough, 2005; De Rubeis and Bagni, 2010), but also mRNA translation (Ceman et al., 2003; Zalfa et al., 2003; Bechara et al., 2009). Furthermore, FMRP was found to influence the microRNA-pathway, thus gaining further control on the expression of its target proteins (reviewed in Kenny and Ceman, 2016).

Inside the nucleus, FMRP localizes to active transcription sites, where it binds to nascent mRNA (Eberhart et al., 1996; Kim et al., 2009) and may even take action in alternative splicing, since G-quartets present in the mRNA of FMRP itself were found to function as exonic splicing enhancers (Didiot et al., 2008). Studies of the transcription and splicing machinery actually revealed brain-region as well as cell type specific alterations in *Fmr1*^−/y^ mice (Derlig et al., 2013) and showed that loss of FMRP results in aberrant transcriptional regulation (Korb et al., 2017). Furthermore, FMRP was found to bind to DNA and to function in DNA damage response (Alpatov et al., 2014) as well as in Heterochromatin organization (Tan et al., 2016). These findings suggest that FMRP may control every step of protein expression from DNA organization to translation.

#### FXS and Group 1 Metabotropic Glutamate Receptors

On the other hand, FMRP itself turned out to be under control of mGluR1/5^9^ signaling cascades (Narayanan et al., 2008), hence allowing for an activity dependent regulation of the mRNA metabolism in various aspects by FMRP. Detailed studies on the function of mGluRs in FXS have lead to the advance of the mGluR-Theory (Huber et al., 2002; Bear et al., 2004; Dolen et al., 2007; Nakamoto et al., 2007): the theory states that FMRP normally acts as a repressor of mRNA translation downstream of group 1 mGluRs, which is released after mGluR activation and thereby induces the translation of proteins required for the expression of LTD. Hence, in the absence of FMRP, persistent and mGluR stimulation independent synthesis of LTD-proteins causes ongoing AMPAR^10^ internalization. The theory further posits that exaggerated mGluR-signaling, perhaps as a consequence of malfunctioning feedback inhibition, is causing many of the symptoms observed in FXS.

There is indeed good evidence for this theory: the pharmacological down-regulation of mGluR5 signaling has been shown to improve a variety of typical symptoms in *Fmr1*^−/y^ mice, including aberrant neuronal morphology, hyperactivity, social behavior, seizure susceptibility and learning and memory (Yan et al., 2005; de Vrij et al., 2008; Levenga et al., 2011; Su et al., 2011; Michalon et al., 2012; Vinueza Veloz et al., 2012; Gantois et al., 2013; de Esch et al., 2015) and so has a genetic reduction of group 1 mGluRs (Dolen et al., 2007; de Esch et al., 2015). One group reported some contradictory findings (Thomas et al., 2011), but the promise of the results prompted clinical trials with mGluR antagonists such as AFQ056 (Novartis) and RO4917523 (Roche) to down-regulate the exaggerated mGluR signaling. Although patients treated with these substances were initially reported to experience some behavioral improvements (Jacquemont et al., 2011), all trials were discontinued during phases IIb/III since the studies did not show any significant improvements in abnormal behaviors compared to placebo (reviewed in Scharf et al., 2015). It has been speculated that cross-reactions with other drugs used to treat FXS patients, the long time of perpetuation in adult patients, irreversible changes during early brain development and difficulties in the outcome measures might have caused the failure, but the underlying reasons have remained unclear.

Remarkably, several targets of FMRP belong to the two major mGluR-signaling cascades controlling the expression of related proteins, the ERK^11^ - and the mTOR^12^ - pathway: ERK, PI3K^13^, PIKE^14^, GSK3^15^ and mTOR are target mRNAs of FMRP (Darnell et al., 2011; Ascano et al., 2012). Both signaling pathways turned out to be exaggerated not only in the hippocampus of *Fmr1*^−/y^ mice (ERK pathway: Hou et al., 2006; Michalon et al., 2012; mTOR pathway: Sharma et al., 2010; Liu Z. H. et al., 2012; Bhattacharya et al., 2016; Choi et al., 2016), but also in humans (Weng et al., 2008; Hoeffer et al., 2012; Wang X. et al., 2012; Kumari et al., 2014; Pellerin et al., 2016).

There has been some dispute about the status of ERK activation in FXS though (mice: Hu et al., 2008; Gross et al., 2010; Osterweil et al., 2010; patients: Yrigollen et al., 2016), but pathway-specific inhibitors of ERK-signaling constantly rescued characteristic deficits in FXS models (Chuang et al., 2005; Osterweil et al., 2010; Wang X. et al., 2012), thus supporting the idea that enhanced mGluR1/5 cascades are causative for some symptoms of the disease. Further studies found that inhibition of ERK-signaling with Metformin (Gantois et al., 2017) or Lovastatin (Osterweil et al., 2013) indeed ameliorates many deficits in *Fmr1*^−/y^ mice. Strikingly though, mTOR inhibition with Rapamycin turned out to induce adverse effects on sleep and social behavior in both, control and *Fmr1*^−/y^ mice (Saré et al., 2018), although positive effects of Rapamycin were reported for the BTBR *T*^+^*Itpr3^tf^*/J mouse model of autism (Burket et al., 2014). In fact, some data even suggest that mTOR inhibition with Rapamycin might cause neurodegeneration (Lin et al., 2013). The hopes are therefore now on clinical trials with Lovastatin (Caku et al., 2014; Pellerin et al., 2016) and Metformin (Dy et al., 2018).

#### Brain Region Specific Mechanisms in FXS

All the above mentioned strategies to amend FXS suffer from the same difficulty though; contrary to drugs, the outlined mechanisms only apply to the hippocampus. There is evidence that the signaling mechanisms of the cortical and hippocampal networks are differentially affected by the loss of FMRP. In cortical synaptosomes of *Fmr1*^−/y^ mice for example, mTOR activity was found normal (Sawicka et al., 2016), whereas in the hippocampus, mTOR signaling is exaggerated (Sharma et al., 2010; Liu Z. H. et al., 2012; Bhattacharya et al., 2016; Choi et al., 2016). Cortical ERK was demonstrated to be erroneously deactivated following mGluR stimulation in *Fmr1*^−/y^ mice (Kim et al., 2008), whereas it was illustrated to be normal (Osterweil et al., 2010) or even over-activated in hippocampal tissue (Hou et al., 2006; Michalon et al., 2012). Remarkably, the study of Sawicka and colleagues showed that ERK signaling in the neocortex of *Fmr1*^−/y^ mice is impinging on ribosomal protein S6, which usually receives input from mTOR, though in this study, cortical ERK activity was found exaggerated.

The data on patients are mostly derived from fibroblasts (Kumari et al., 2014; Yrigollen et al., 2016) or thrombocytes (Weng et al., 2008; Pellerin et al., 2016), making conclusions on the characteristics of specific brain regions difficult. Using post-mortem tissue, one study detected no differences in ERK activation in the frontal lobe between FXS patients and controls (Hoeffer et al., 2012), while another one found increased levels of phosphorylated ERK in the frontal cortex of patients (Wang X. et al., 2012). Despite the unclear status of ERK activation in the cortex, these studies suggest that mGluR1/5 signaling pathways are functioning differently in the cortex and the hippocampus. In line with this notion, a recent study of mTOR activity and exercise demonstrated that the activation status of mTOR is depending on the brain region, the cell type (neuron or glia) and the type of exercise (sedentary, voluntary or forced; Lloyd et al., 2017).

##### Long term potentiation

Further support for the relevance of brain region specific mechanisms in FXS comes from a variety of studies on synaptic plasticity. Synaptic plasticity is mostly investigated by the induction of LTP^16^ or LTD^17^, two paradigms which are considered cellular models of learning and memory (reviewed in Kandel, 2001; Bliss et al., 2003; Neves et al., 2008). Both, LTP and LTD, ultimately depend on the modulation of synaptic signaling and have been studied intensively in *Fmr1*^−/y^ mice using manifold induction protocols (please see Table 3 for more details).

**Table 3:** LTP protocols used in different studies.

Study	Stimulation	Recording	Study	Stimulation	Recording
Auerbach and Bear (2010)	*Hippocampus*: 1 s 100 Hz tetanus stimulation	Field recordings	Bostrom et al. (2015)	*Hippocampus*, CA1 and *dentate gyrus*: 4 trains of 50 pulses at 100 Hz, 30 s apart	Field recordings
Chen et al. (2014)	*Anterior cingulate cortex*: five trains of bursts with four pulses at 100 Hz and 200 ms interval; repeated five times at intervals of 10 s	MED64 probe (array)	Godfraind et al. (1996)	*Hippocampus*: data not available	Field recordings
Harlow et al. (2010)	*Somatosensory cortex*: pairing of 100 stimuli at 1 Hz with postsynaptic depolarization to 0 mV	Whole cell recordings	Hayashi et al. (2007)	*Cortex*: eight bursts (each four pulses at 100 Hz) every 200 ms	Field recordings
Hu et al. (2008)	*Hippocampus*: pairing of 200 pulses at 2 Hz at −5 mV within 5 min after formation of whole-cell configuration	Whole-cell recordings	Koga et al. (2015)	*Anterior cingulate cortex*: induction of pre-LTP with repetitive low-frequency stimulation at 2 Hz for 2 min	Whole-cell recordings (neurons), multielectrode array (slices)
Larson et al. (2005)	*Anterior piriform cortex*: 10 bursts at 100 Hz with four pulses repeated in 200 ms intervals, *hippocampus*: two pathways, one by five theta bursts, the other by 10 theta bursts	Field recordings	Lee et al. (2011)	*Hippocampus*: five theta burst stimuli	Field recording
Li et al. (2002)	*Hippocampus*: single tetanic train of 100 Hz, 1 s duration at maximal intensity, *cortex*: three tetanic trains of 200 Hz, 1 s with an interval of 10 min	Field recordings	Martin H. G. S. et al. (2016)	*Prefrontal Cortex*: five trains of burst with four pulses at 100 Hz and 200 ms interval, repeated four times at intervals of 10 s	Whole-cell recordings
Padmashri et al. (2013)	*Motor cortex*: chemical LTP via bath application of bicuculline (6.3 μm) for 3 min, followed by forskolin (50 μm) and the phosphodiesterase inhibitor rolipram (0.1 μm) in Mg^2+^-free ACSF for 15 min	Field recordings	Paradee et al. (1999)	*Hippocampus*: stimulation 1x every 30 s for 20 min for baseline response, followed by induction of L-LTP by three trains (10 bursts at 5 Hz, each burst consisting of a 40 ms burst at 100 Hz) of theta bursts, 1 min apart	Field recordings
Shang et al. (2009)	*Hippocampus*: stimulation intensity adjusted so that a half-maximal fEPSP was elicited	Field recording, whole-cell recording	Wilson and Cox (2007)	*Neocortex*: three trains of 100 Hz, 1-s duration at 5 min intervals	Field recordings
Xu et al. (2012a)	*Prefrontal cortex*: 80 pulses at 2 Hz, and then paired with postsynaptic depolarization at + 30 mV	Whole-cell recordings	Xu et al. (2012b)	*Anterior cingulate cortex*: 80 pulses at 2 Hz paired with postsynaptic depolarization at +30 mV	Whole-cell recordings
Yang et al. (2014)	*Auditory cortex*: three repetitions of 100-Hz stimulation of 1-s duration.	Field recordings	Zhang et al. (2009)	*Hippocampus*: L-LTP induced by four 1 s trains of 100 Hz with a 5 min interval	Field recordings
Zhao et al. (2005)	*Anterior cingulate cortex*: 80 pulses at 2 Hz paired with postsynaptic depolarization at +30 mV	Whole-cell recordings			

*Literature providing only general information is not included. The data illustrates the variety of induction protocols used to study LTP*.

While some protocols have led to the discovery of disturbances in certain forms and aspects of hippocampal LTP (Hu et al., 2008; Shang et al., 2009; Lee et al., 2011), most examinations found the expression of hippocampal LTP in *Fmr1*^−/y^ mice to be normal (Godfraind et al., 1996; Paradee et al., 1999; Li et al., 2002; Larson et al., 2005; Zhang et al., 2009; Auerbach and Bear, 2010). Two of these studies also investigated cortical LTP: while they observed no abnormalities in hippocampal LTP, both detected significantly impaired LTP in the cortex of *Fmr1*^−/y^ mice (Li et al., 2002; Larson et al., 2005). The result is line with several studies demonstrating defective LTP in different regions of the cortex (Zhao et al., 2005; Hayashi et al., 2007; Wilson and Cox, 2007; Harlow et al., 2010; Xu et al., 2012b; Padmashri et al., 2013; Yang et al., 2014; Koga et al., 2015).

LTP includes two distinct phases, an early phase (E-LTP), which does not require protein synthesis, and a late phase (L-LTP), which depends on protein synthesis and gene expression (Frey et al., 1993; Abel et al., 1997, reviewed in Kandel, 2001, 2009). While L-LTP is unaffected in the hippocampus of *Fmr1*^−/y^ mice (Paradee et al., 1999; Zhang et al., 2009), it is blocked in the cingulate cortex (Chen et al., 2014). Since pharmacological inhibition of mGluR5 or GSK3 rescued L-LTP in the cingulate cortex of *Fmr1*^−/y^ mice, these results show that exaggerated mGluR signaling is involved. Indeed, two other studies also reported rescues of cortical LTP based on mGluR1/5 antagonists (Xu et al., 2012a; Martin H. G. S. et al., 2016), but the effects of mGluR5 or GSK3 inhibition on (L-)LTP in the hippocampus were never investigated although hippocampal L-LTP is known to depend on mGluR1/5 activation (Riedel and Reymann, 1996; Francesconi et al., 2004; Neyman and Manahan-Vaughan, 2008; Fan, 2013).

The differences between the hippocampus and the cortex are further emphasized by the fact that age-related deficits in LTP were discovered specifically in the cortex (Larson et al., 2005; Martin H. G. S. et al., 2016). While *Fmr1*^−/y^ mice older than 6 months displayed significant defects in the expression of cortical LTP, the mice never displayed any impairment in hippocampal LTP. In fact, Bostrom et al. (2015) even demonstrated differences within the hippocampus itself: while the loss of FMRP caused impairments in NMDAR^18^-dependent LTP in the dentate gyrus, NMDAR-LTP was found normal in the CA1^19^ region of the hippocampus.

##### Long term depression

The most prominent and best studied plasticity model in FXS is mGluR-LTD, a type of LTD that depends on the activation of mGluR1/5, protein synthesis and the internalization of AMPA receptors. However, while numerous studies demonstrated enhanced mGluR-LTD in the hippocampus of *Fmr1*^−/y^ mice (Huber et al., 2002; Hou et al., 2006; Nosyreva and Huber, 2006; Volk et al., 2007; Park et al., 2008; Ronesi and Huber, 2008; Zhang et al., 2009; Auerbach and Bear, 2010; Choi et al., 2011; Bhattacharya et al., 2012; Michalon et al., 2012; Niere et al., 2012; Costa et al., 2015; Toft et al., 2016; Thomson et al., 2017), nobody studied mGluR-LTD in the cortex yet. There is evidence though that cortical mGluR-LTD is existing (reviewed in Kang and Kaang, 2016). Using a protocol to induce spike-time-dependent plasticity (STD-LTP or STD-LTD) in the neocortex of *Fmr1*^−/y^ mice, Desai and colleagues found no impairment in LTD, but a significant reduction in LTP (Desai et al., 2006). Experiments with MPEP^20^, a mGluR5 antagonist, revealed that cortical STD-LTP is not depending on mGluR5 activation, whereas cortical STD-LTD is. Remarkably, the application of anisomycin, an inhibitor of protein synthesis, revealed that cortical STD-LTD does not require protein synthesis, thus suggesting that FMRP is not necessary for cortical STD-LTD despite the dependence on mGluR5. Sadly though, STD-LTD has not been investigated in the hippocampus yet.

It is worth noting that the biological relevance of mGluR-LTD has recently been questioned, arguing that mGluR-LTD in the absence of previous LTP is artificial (discussed in Jones, 2017). Nonetheless, since more than 15 years of mGluR-LTD research in *Fmr1*^−/y^ mice did not provide any evidence for abnormal mGluR-LTD in the cortex, it seems likely that the mechanisms underlying this form of plasticity differ among brain regions.

##### Keeping the balance

FXS is associated with a vast misregulation of protein expression, not only in the context of mGluR signaling, but also with respect to proteins regulating other aspects of neuronal excitability such as Calmodulin or Neuroligin for instance (Liao et al., 2008; Matic et al., 2014; Kalinowska et al., 2015; Tang et al., 2015). Although it is not clear yet whether abnormal mGluR-LTD directly impacts on the balance of excitatory and inhibitory activity in neuronal networks (E/I balance), mGluR signaling is able to alter the excitability of neurons by increasing the intrinsic conductance (Bianchi et al., 2009; Tang and Alger, 2015). Furthermore, FMRP itself may bind to ion channels such as Calcium, Slack^21^ and BK^22^ channels, thereby providing an additional level of FMRP mediated control (Brown et al., 2010; Deng et al., 2013; Ferron et al., 2014).

Deviations in the balance of excitation and inhibition have been associated with seizure activity, hypersensitivity and cognitive deficits in several ASDs and animal models (reviewed in Frye et al., 2016; Uzunova et al., 2016; Lee et al., 2017; e.g., Orekhova et al., 2008; Tebartz van Elst et al., 2014; Robertson et al., 2016), including *Fmr1*^−/y^ mice (e.g., D’Hulst et al., 2006; Zhong et al., 2009; Dahlhaus and El-Husseini, 2010; Aguilar-Valles et al., 2015; Deng and Klyachko, 2016). Using these animals, in particular neocortical circuits have been shown to experience enhanced excitation (Gibson et al., 2008; Goncalves et al., 2013; Patel et al., 2013; Zhang Y. et al., 2014; Westmark et al., 2016), partially due to inhibitory deficits (Selby et al., 2007) and potentially supported by inhibitory dysfunctions in the cortico-hippocampal pathway and inhibitory defects in feed-forward circuits (Wahlstrom-Helgren and Klyachko, 2015).

Indeed, not only glutamatergic signaling has been demonstrated to be altered in *Fmr1*^−/y^ mice, but also gabaergic and dopaminergic mechanisms have been found malfunctioning. Studies showed that *Fmr1*^−/y^ mice experience diminished GABAa/b^23^ receptor expression, reduced GABA release, decreased dopamine receptor expression and malfunctioning interneurons (D’Hulst et al., 2006; Selby et al., 2007; Pacey et al., 2011b; Paluszkiewicz et al., 2011; Henderson et al., 2012; Heulens et al., 2012; Patel et al., 2013; Paul et al., 2013; Berzhanskaya et al., 2016a; Kang et al., 2017). Deficiencies in inhibitory conductance are therefore characteristic to many circuits of the murine FXS brain including circuits of the striatum, amygdala, hippocampus, subiculum and the somatosensory as well as the prefrontal cortex (Centonze et al., 2008; Curia et al., 2009; Olmos-Serrano et al., 2010; Paluszkiewicz et al., 2011; Vislay et al., 2013; Martin et al., 2014; Sabanov et al., 2016). In addition, the loss of interactions between FMRP and BK channels causes uncontrolled Glutamate release, altered action potential waveforms and exaggerated excitability (Zhang Y. et al., 2014; Myrick et al., 2015; Deng and Klyachko, 2016).

Recent research indicates that the alterations in the E/I balance may include brain region and circuit specific mechanisms. For instance, while dendrites of hippocampal neurons from *Fmr1*^−/y^ mice display increased HCN1-channel^24^ expression and reduced input resistance (Brager et al., 2012), dendrites of cortical layer 5 neurons show the opposite (Zhang Y. et al., 2014). The intrinsic membrane excitability of cortical layer 4 excitatory neurons is exaggerated (Gibson et al., 2008), while that of excitatory hippocampal neurons is normal (Deng et al., 2013; Luque et al., 2017). Hippocampal neurons demonstrate significantly longer action potential durations and higher firing frequencies in the absence of FMRP than under normal conditions (Luque et al., 2017), whereas layer 2/3 neurons in the prefontral cortex present significantly narrower and taller action potentials in *Fmr1*^−/y^ mice than in their wildtype litter mates (Routh et al., 2017). Although both alterations indicate enhanced excitability, the sharpened action potentials observed the cortex of *Fmr1*^−/y^ mice are in contrast with the broadened action potentials seen in the hippocampus. Since two studies even demonstrated increased inhibition early in development (Berzhanskaya et al., 2016b; Truszkowski et al., 2016), the data show that the alterations in neuronal activity depend on the specific circuit, aspect and age.

Despite the high complexity of the system, several studies were able to rescue the aberrant neuronal activity based on restorations of mGluR1/5 signaling (Meredith et al., 2011; Ronesi et al., 2012; Westmark et al., 2016; Aloisi et al., 2017), GABA signaling (Olmos-Serrano et al., 2010, 2011; Martin B. S. et al., 2016; Kang et al., 2017) or ion channel function (Zhang Y. et al., 2014; Deng and Klyachko, 2016; Aloisi et al., 2017). In doing so, phenotypes such as epileptiform activity (Ronesi et al., 2012; Zhang Y. et al., 2014; Deng and Klyachko, 2016; Westmark et al., 2016), hyperactivity (Olmos-Serrano et al., 2011; Ronesi et al., 2012) and hypersensitivity (Zhang Y. et al., 2014) were normalized. A few studies have also investigated network oscillations in FXS model animals (Gibson et al., 2008; Goncalves et al., 2013; Rotschafer and Razak, 2013; Radwan et al., 2016; Westmark et al., 2016; Berzhanskaya et al., 2017). In these experiments, cortical neurons displayed increased synchrony in their activity as well as a threefold higher firing rate during Up states (Gibson et al., 2008; Goncalves et al., 2013; Westmark et al., 2016), increased high-frequency as well as reduced low-frequency power during rest (Berzhanskaya et al., 2017) and elevated responses to auditory stimuli (Rotschafer and Razak, 2013).

The findings support the theory that neuronal hyperexcitability is a leading cause for many symptoms of FXS and fit well with the Intense World Theory of Autism (reviewed in Markram and Markram, 2010), which posits that hyperactive micro-networks cause many of the cognitive deficits characteristic to ASDs, in particular hypersensitivity, hyperattention, hyperemotionality and seizure susceptibility. Although the studies of oscillatory dynamics in FXS patients currently available mostly confirm the idea of imbalanced circuit activity (Castrén et al., 2003; Dalton et al., 2008; Holsen et al., 2008; Van der Molen and Van der Molen, 2013; Van der Molen et al., 2014; Wang et al., 2017), not all of them are in favor of the theory: two studies detected decreased activity, one in prefrontal regions and one in the fusiform gyrus (Dalton et al., 2008; Holsen et al., 2008), while a third study found intracortical inhibition in FXS patients to be normal (Oberman et al., 2010).

## From Mice to Men

### Literature Summary

Most rescues reported from *Fmr1*^−/y^ mice are based on investigations of the hippocampus (Table 4). Some studies included data on further brain regions or transferred approaches to cortical regions, analyzing different aspects of mGluR signaling for example; however, only 4 studies were found that focussed on the cortex in terms of their strategy and their experiments. No studies could be identified that are reporting behavioral rescues based on mechanisms characteristic to the amygdala, cerebellum, striatum or any other brain region in specific, although the available data argue for different deficits in different brain regions (see for instance Chen et al., 2014; Bostrom et al., 2015; Sawicka et al., 2016; Lloyd et al., 2017) and despite the fact that rescues of neuronal activity have been reported for the amygdala (Olmos-Serrano et al., 2010, 2011; Suvrathan et al., 2010; Martin B. S. et al., 2016). These findings show that there is a significant imbalance between the current hippocampus centered investigations and the multifarious mechanisms found in the brain of wildtype as well as FXS model animals.

**Table 4:** Brain-region bias in FXS research.

	Rescue of behavior	Rescue of neuronal function only
Hippocampus based strategies and/or studies	Yan et al. (2005), Dolen et al. (2007), de Vrij et al. (2008), Gross et al. (2010, 2015a), Levenga et al. (2011), Westmark et al. (2011), Bhattacharya et al. (2012), Goebel-Goody et al. (2012), Guo et al. (2012), Liu Z. H. et al. (2012), Michalon et al. (2012), Ronesi et al. (2012), Vinueza Veloz et al. (2012), Chen et al. (2013), Gantois et al. (2013), Osterweil et al. (2013), Udagawa et al. (2013), Boda et al. (2014), Franklin et al. (2014), Hébert et al. (2014), Sidhu et al. (2014), Sun et al. (2014, 2016), Tian et al. (2015), de Esch et al. (2015), Aloisi et al. (2017), Martinez and Tejada-Simon (2017), Pardo et al. (2017) and Thomson et al. (2017) total: 30	Lauterborn et al. (2007), Nakamoto et al. (2007), Zeier et al. (2009), Choi et al. (2011), Gross et al. (2011), Meredith et al. (2011), Costa et al. (2012, 2015), Deng et al. (2013), Bostrom et al. (2015), Choi C. H. et al. (2015), Ghilan et al. (2015), Tang and Alger (2015), Zhao W. et al. (2015), Deng and Klyachko (2016), Toft et al. (2016), Westmark et al. (2016) and Yau et al. (2016) total: 18
Studies including data on several brain regions	Yuskaitis et al. (2010), Liu et al. (2011), Pacey et al. (2011a), Ronesi et al. (2012), Xu et al. (2012a), Gkogkas et al. (2014), Lim et al. (2014), Braat et al. (2015), Bhattacharya et al. (2016) and Li et al. (2016) total: 10	
Cortex based strategies and/or studies	Hayashi et al. (2007), Dolan et al. (2013), Gross et al. (2015c) and Yang et al. (2015) total: 4	Henderson et al. (2012), Kim et al. (2013), Chen et al. (2014) and Lovelace et al. (2016), Westmark et al. (2016) total: 5
Amygdala based strategies and/or studies	none	Olmos-Serrano et al. (2010, 2011), Suvrathan et al. (2010) and Martin B. S. et al. (2016) total: 4
Brain regions not specified/studied	Veeraragavan et al. (2011), Heulens et al. (2012), Gholizadeh et al. (2014) and Pietropaolo et al. (2014) total number: 4	

*The table lists all publications that were identified on pubmed or PMC in October 2017, and found to report at least functional rescues of symptoms related to FXS using murine model systems. Studies reporting only morphological rescues were not included. The data show that there is a strong bias toward investigations of hippocampal functions in the literature*.

### The Cortex, the Hippocampus and FXS

Problems in translating findings from mice to men are a common phenomenon in brain research. A major difficulty arises from the development of the cerebral cortex in primates and in particular in humans. Recent research revealed that the cognitive performance of vertebrates is best reflected by a combination of the number of neurons, neuron density and axonal conduction velocity in the cortex (reviewed in Dicke and Roth, 2016). In this regard, mice are not well set, since their cerebral cortex exhibits only a relatively low density of neurons: if mice would have a brain the size of a human, their cerebral cortex would contain 2 billion neurons, whereas the human cerebral cortex does in fact have 16 billion (cp. Herculano-Houzel, 2009). However, neuron density is not the only disadvantage mice are confronted with:

- the neocortex constitutes 80% of the human brain (Azevedo et al., 2009), but only 40% of a mouse brain (reviewed in Herculano-Houzel, 2009)
- while mice have about 20 different cortical areas, humans have more than 200 (Kaas, 1989, 2011; Changizi and Shimojo, 2005)
- most cortical areas in mice are related to sensory and motor functions, whereas the majority of the human areas function in association (reviewed in Buckner and Krienen, 2013)
- some parts of the human neocortex are specifically enlarged compared to the rest of the neocortex:

- the prefrontal cortex and area 10 (Semendeferi et al., 2002, 2011), which is important to higher cognitive functions,
- the insula, which usually functions in sensory information processing, but has new areas in humans that are functioning in empathy and social awareness (reviewed in Keysers et al., 2010) and
- the posterior parietal cortex (reviewed in Orban et al., 2006), which is important to planning, imitation and the highly skilled use of tools.

- by contrast, the primary sensory and motor areas have maintained their relative sizes during the evolution from mice to men (Hill et al., 2010; Preuss, 2011)
- several cell types found in the human brain do not exist in mice (reviewed in Buckner and Krienen, 2013).

In line with these findings, rodents perform relatively poor in behavioral tasks when compared to monkeys possessing similar sized brains (e.g., capybara (Macdonald, 1981) and capuchin monkey (Anderson et al., 2013; De Moraes et al., 2014; Takahashi M. et al., 2015)), thus suggesting that primate brains have properties, which are significantly different from those of rodents.

This notion is emphasized by an elegant study of Han and colleagues (Han et al., 2013), who showed that human glia cells are much more competent than murine glia cells when it comes to supporting brain functions: replacing the glia of mice with human cells, they observed that the engrafted mice were able to propagate calcium signals three times faster than allografted mice, exhibited sharply enhanced LTPs and performed excellent in cognition tests. Indeed, astrocytes are able to coordinate and modulate neural signal transmission (reviewed in Allen, 2014; Allen and Eroglu, 2017), but human astrocytes differ from their murine counterparts in that human astrocytes are larger, more complex and more diverse, have much more synaptic contacts and are more efficient in calcium signaling (Andriezen, 1893; Colombo, 1996; Colombo et al., 1997, 2000; Reisin and Colombo, 2002; Oberheim et al., 2009).

Remarkably, this is particularly true for the cerebral cortex: there are at least four morphologically distinct astrocyte classes within the primate cortex, as compared to two in rodents. The two novel classes are interlaminar astrocytes and varicose projection astrocytes (Andriezen, 1893, reviewed in Vasile et al., 2017). Varicose projection astrocytes have hitherto been observed only in cortical layers 5–6 of humans and chimpanzees, but the human cells are more complex. By contrast, interlaminar astrocytes reside in upper cortical layers and extend long processes to cortical layers 3 and 4 (Colombo, 1996; Colombo et al., 1997; Reisin and Colombo, 2002; Korzhevskii et al., 2005; Oberheim et al., 2009). Although interlaminar astrocytes are present in both, monkeys and men, humans have higher numbers of interlaminar astrocytes. Despite the fact that more than 120 years have passed since their initial description, the role of these primate-specific astrocytes is still elusive.

Nonetheless, astroglia seem to have a role in FXS: though FMRP is predominantly expressed in neurons at all ages, it is also seen in oligodendrocyte precursor cells and astrocytes. While neurons display a gradual decrease in FMRP expression during development (Davidovic et al., 2011; Bonaccorso et al., 2015), astrocytes and oligodendrocyte precursor cells mainly express FMRP during early and mid-postnatal stages of brain maturation (Wang et al., 2004; Gholizadeh et al., 2015), thus suggesting a role for glia and FMRP in development. Indeed, it was shown that FMRP deficient astrocytes cause developmental delays in dendrite maturation and synaptic protein expression of hippocampal wildtype neurons (Jacobs et al., 2010, 2016), whereas normal astrocytes prevent abnormal dendritic development in hippocampal FXS neurons (Jacobs and Doering, 2010).

Investigations on the underlying mechanisms are also still in their infancy. In line with the role of FMRP in regulating protein expression, it was recently shown that the astrocyte-secreted factors Hevin^25^ and SPARC^26^, which function to control excitatory synapse development, display abnormal expression patterns in hippocampal and cortical tissues from *Fmr1*^−/y^ mice (Wallingford et al., 2017): while hippocampal Hevin expression is gradually increasing from a reduced expression at P7^27^ to elevated levels at P21, Hevin expression in the cortex displays only a transient increase at P14. SPARC, on the other hand, shows a modest decrease at P7 and P14 in the cortex, but no abnormalities in the hippocampus. Emphasizing the relevance of this data, Cheng and colleagues were able to demonstrate that astrocyte-conditioned medium is sufficient to prevent morphological deficits in hippocampal neurons cultured from FXS model mice (Cheng et al., 2016). Furthermore, Higashimori and colleagues showed that a selective knock-out of astroglial FMRP in mice modestly increases spine density and size in cortical neurons, whereas a selective re-expression is able to attenuate the abnormal spine morphology characteristic for FXS (Higashimori et al., 2016). The group also described a normalization of the FXS-typical gain in body weight, but no data on cognitive performances were reported. Studies with human astroglia would be desirable, but are currently missing.

The expansion of neocortical areas in primates caused a profound rewiring of the cortex. As a consequence, the human association cortex lacks the strict hierarchical organization of circuits seen in rodents, although certain projections follow the canonical form (e.g., parieto-prefrontal projections), but is instead characterized by multiple, large-scale distributed and highly interwoven networks termed non-canonical circuits. These circuits are highly active during cognitive performances (reviewed in Goldman-Rakic, 1988; Buckner and Krienen, 2013; Margulies, 2017).

Notably, not only the internal pathways of the cortex changed during the evolution from mice to men, also the wiring between the cortex and the hippocampus was modified: in men, the hippocampus preferentially connects to cortical association networks, whereas in mice, it preferentially associates with sensory networks (Bergmann et al., 2016). Although there are still two conserved parallel pathways between the cortex and the hippocampus in mice and men, which transfer object and context related information (reviewed in Ranganath and Ritchey, 2012), the finding of Bergmann and colleagues is of quiet some significance since cortical-hippocampal pathways are required for important brain functions including spatial working memory (studies in rodents and humans; reviewed in Sigurdsson and Duvarci, 2016), long-term memory (studies in rodents, primates and humans; reviewed in Sigurdsson and Duvarci, 2016), motivation and emotion (studies in rodents; reviewed in Sigurdsson and Duvarci, 2016), and social recognition (studies in rodents and humans; reviewed in Bicks et al., 2015). Indeed, weaknesses in working memory performance, in particular when requiring abstract item reasoning, are characteristic to FXS patients (Munir et al., 2000; Cornish et al., 2001; Kwon et al., 2001; Ornstein et al., 2008; Baker et al., 2011; Wang et al., 2013), but not to model mice (Leach et al., 2016). While *Fmr1*^−/y^ mice perform as well as their wildtype littermates even when their working memory is significantly challenged (Leach et al., 2016), individuals with FXS are unable to modulate activation of the prefrontal and parietal cortex in response to an increasing working memory load (Kwon et al., 2001), implying a lack of circuit control. Indeed, a fMRI^28^ study showed that decreased levels of FMRP correlate with decreases in parahippocampal activation and reduced connectivity between the hippocampus and the prefrontal cortex in patients (Wang J. M. et al., 2012).

Emotional and social difficulties are frequently observed in individuals with FXS as well (Cohen et al., 1988; Mazzocco et al., 1994; Hall et al., 2009; Cordeiro et al., 2011; Kim et al., 2014), but the data on mice are inconsistent (reviewed in Kazdoba et al., 2014). Similar to the impairments observed in working memory performance, social deficits were associated with impairments in the activation of prefrontal regions in individuals with FXS (Holsen et al., 2008). Taken together, these data demonstrate that the cortical and cortical-hippocampal circuits, which characterize the human brain, are critical to FXS and cannot be appropriately modeled in mice. Even though mice may perform the same tasks as men, it is obligatory to investigate whether they also do it in the same way.

In the hippocampus, the situation is slightly different. Current research does not support major differences in the structure and function of hippocampi from mice and men (reviewed in Clark and Squire, 2013). A study comparing human and murine spatial navigation showed that both species use the same strategies (Eilam, 2014) to solve this basic and evolutionary old task. Spatial navigation in mammals is assumed to rely on hippocampal place cells, entorhinal grid cells (reviewed in Moser et al., 2008) and hippocampal theta oscillations, albeit neuronal firing in distant brain regions such as the somatosensory or prefrontal cortex is phase-locked to hippocampal theta oscillations (reviewed in Jacobs, 2013). Interestingly, the frequencies of these theta oscillations differ among species: while rats typically display oscillations at 4–8 Hz, human theta oscillations have a frequency of approximately 1–4 Hz (reviewed in Jacobs, 2013).

In line with this data, both, *Fmr1*^−/y^ mice as well as FXS patients were found to show elevated error rates in the Hebb-Williams maze (MacLeod et al., 2010), a test that analyses spatial memory performance in humans and rodents under comparable conditions. In a follow-up study, the group was able to rescue the deficit in *Fmr1*^−/y^ mice using MPEP, an antagonist of mGluR5 (Gandhi et al., 2014), however, contrary to humans, mice did not show increased latencies, implying differences between FXS individuals and model mice. The authors suggest that the discrepancy might arise from differences in the presentation of the maze, that is real vs. virtual, but since the human control group faced the same difficulty, this would rather argue for a different navigation strategy in FXS individuals, a strategy, that relies more on real information, such as obtained from walking, touching or smelling for instance. Indeed, difficulties in abstract thinking are characteristic for FXS patients, but not for model mice, which do as well as their wildtype litter mates in tests employing touch screens (cp. the review of Huddleston et al., 2014 and Leach et al., 2016). Since the Hebb-Williams maze is rarely used for rodents and was not employed to measure outcomes in clinical trials, conclusions are difficult to draw, in particular in the light of the role of cortical connections in human cognition and of the mixed results obtained from mice (reviewed in Kazdoba et al., 2014). However, even under conditions clearly favoring a translation between mice and men, such as in spatial navigation paradigms, the rodent model apparently reflects the situation in humans only partially.

### Alternatives

Poor translatability represents indeed a major issue in most preclinical brain research. In order to overcome this obstacle, touch screen paradigms have recently been developed for rodents, which center on the Cambridge Neuropsychological Test Automated Battery for humans (reviewed in Horner et al., 2013; Hvoslef-Eide et al., 2016; Kangas and Bergman, 2017). Although these assays analyze specific cognitive abilities known to be affected in patients and achieve a high degree of standardization as well as high throughputs, *Fmr1*^−/y^ mice failed to recapitulate the working memory impairments characteristic to individuals with FXS in corresponding tests even when their cognitive performance was challenged in a non-match to position task with increasing delays (Leach et al., 2016). The reasons are elusive. It is possible that the excessive pre-training as well as the correction trials required for mice affect the interpretation of results. Also, since working memory relies on the prefrontal cortex, differences in the cognitive processing can be expected, but studies are missing. More research is required to validate the neurocognitive mechanisms underlying each behavioral paradigm in our species of interest and to identify routes as well as boundaries of translation across species.

The outlined data illustrate that the current FXS mouse models fail to mirror important genetic as well as behavioral aspects of FXS, probably due to the evolutionary distance of mice and men. To address this issue, two rat models were recently developed, *Fmr1* KO^29^ rats (Engineer et al., 2014; Till et al., 2015; Berzhanskaya et al., 2016b; Kenkel et al., 2016) and *Fmr1* exon4 KO rats (Tian et al., 2017). The investigations revealed that the new model animals indeed recapitulate many features of the murine model, including enhanced basal protein synthesis, exaggerated hippocampal mGluR-LTD, elevated dendritic spine densities and cortical hyper-excitability (Till et al., 2015; Berzhanskaya et al., 2016b; Tian et al., 2017). However and much to a surprise, the rats neither reflect the behavioral phenotype of mice very well (Till et al., 2015), nor reproduce the symptoms of FXS patients any better than the murine model: although the animals demonstrated deficits in hippocampus-dependent learning (Till et al., 2015; Tian et al., 2017), they failed to display defects in spatial reference memory and reversal learning (Till et al., 2015), thereby contrasting not only the majority of data obtained from *Fmr1*^−/y^ mice (D’Hooge et al., 1997; Paradee et al., 1999; Van Dam et al., 2000; Baker et al., 2010, reviewed in Kazdoba et al., 2014), but also the weak performance of FXS patients in tasks requiring the solution of new problems (Dykens et al., 1987; Maes et al., 1994; Loesch et al., 2004; Lewis et al., 2006; Van der Molen et al., 2010). Instead, these results rather imply that there is a clade-dependent component in the behavioral phenotype observed in FXS, causing the same molecular and cellular changes to give rise to different behavioral phenotypes. Given the high number of proteins affected by the loss of FMRP, numerous options exist, through which the genetic background may impact on the symptoms of the disorder. It is indeed long known that the specific phenotype of FXS model mice varies significantly among strains (reviewed in Bernardet and Crusio, 2006; Kazdoba et al., 2014). Further studies would be needed to characterize the performance of FXS model rats in cognition as well as sociality in more detail and to explain certain discrepancies between the two rat models (see Till et al., 2015; Tian et al., 2017), but it might not be worth the effort.

Considering the evolution of cognition in primates, the logical model organism for human brain function would be a closely related primate. Although virus-mediated transgenesis (Sasaki et al., 2009; Liu et al., 2016; Park et al., 2016) or ZFN^30^/TALEN^31^-driven genome editing (Sato et al., 2016) have been used to generate primate models, the invention of Crispr-mediated genome editing and its successful employment in primates (Niu et al., 2014; Chen et al., 2015; Tu et al., 2017; Zhao et al., 2017; Zuo et al., 2017) and even in (nonviable) human embryos (Liang P. et al., 2015; Tang et al., 2017) have brought this option within much closer reach. Compared to rodents, primates have several advantages when it comes to modeling cognition (for a comprehensive review, please also see “Why primate models matter”; Phillips et al., 2014), in particular with respect to ASDs:

- the cell types and circuits seen in primates are more similar to those found in humans, which is relevant to many cognitive tasks, but particularly important to spatial working memory and social recognition (see Hopkins, 2013; Frey et al., 2014; Neubert et al., 2014; Morecraft et al., 2015; Wilson et al., 2015a; and the section: “The Cortex, the Hippocampus and FXS”)
- the prolonged prenatal development of the cortex, which is characteristic to humans and primates, is not present in rodents, hampering neuro-developmental studies in rodents
- group living, co-operative behavior and cultural intelligence are much more sophisticated in primates (cp. the section: “The Cortex, the Hippocampus and FXS” and Decasien et al., 2017; Street et al., 2017)
- the basic communication features characteristic to human language are already present in primates, including the ability to utilize symbolization, basic semantic representation, categorical representation and rudimentary grammar (Moore et al., 2003, 2005, 2006; Joly et al., 2012; Morrill et al., 2012; Ghazanfar et al., 2013; Wilson et al., 2013, 2015b)
- many tests developed to analyze cognition in humans can easily be adapted for primates, e.g., eye-tracking to study abnormal gaze (Machado and Nelson, 2011; Rosati et al., 2016), a typical symptom of autism spectrum disorders, or computerized cognition tests (Spinelli et al., 2004; Harris et al., 2007; Barner et al., 2008; Diester and Nieder, 2010; Jones et al., 2010; Takemoto et al., 2011; Verrico et al., 2011; Beran et al., 2012, 2015; Brosnan et al., 2012; Evans and Beran, 2012; Basile and Hampton, 2013; Klein et al., 2013; Bramlett-Parker and Washburn, 2016; Oikonomidis et al., 2017), thereby facilitating the translation of results

Taken together, these studies show that many features characterizing human behavior and typically affected in ASDs, such as communication capabilities and social skills or abstract thinking for instance, are represented in primates. A primate model would hence facilitate investigations addressing core symptoms of the autism phenotype, which are absent or only rudimentary developed in rodents.

There is another important feature of the disease, which cannot be modeled in mice: hypermethylation. Contrary to mice, in which most CpG-islands have apparently eroded during evolution (Aïssani and Bernardi, 1991; Matsuo et al., 1993), primates show methylation patterns highly similar to those of humans. Approximately 90% of the methylation patterns are in fact conserved between men and chimpanzees (Martin et al., 2011; Molaro et al., 2011; Pai et al., 2011), in particular at CpG-islands and promoter regions (Illingworth et al., 2010; Hernando-Herraez et al., 2013, 2015; Long et al., 2013). However, when comparing those genes that are differentially methylated, human genes turned out to exhibit lower levels of promoter methylation than genes from chimpanzees (Gama-Sosa et al., 1983; Zeng et al., 2012), suggesting that gene expression control is less strict in humans. Since a disproportional high number of differentially methylated genes is associated with human diseases (Zeng et al., 2012; Fukuda et al., 2013; Hernando-Herraez et al., 2013), it seems possible that the reduced control of certain genes, which finally made us human, also makes us more susceptible to certain pathologies such as senescence and Alzheimer’s disease, disorders, which are not present in our closest relatives, the great apes (reviewed in Finch and Austad, 2015; Lowenstine et al., 2016). Notably, though some of the genes shown to be differentially methylated in humans are known for their association with ASDs, such as GABAa and GABAb receptors for instance (Zeng et al., 2012; Hernando-Herraez et al., 2013), *FMR1* was not associated with human-specific methylation patterns yet.

Despite these promising premises, primate models also have some disadvantages when compared to rodent models: Working with primates, in particular with great apes, is expensive by all means, it requires a lot of training and experience, bears the risk of transmitting diseases and the longevity of great apes hampers investigations of senescence. Significant n-numbers are often hard to achieve. Hence, primate species which are small, relatively short lived and easy to keep could help to overcome these obstacles.

The evolutionary next closest alternatives to great apes are old world monkeys. Consisting of about 160 species, old world monkeys represent the largest group of primates. Among them are the macaque monkeys, which include two species, that have been used in science for a long time, the crab-eating (alias: cynomolgus; *M. fascicularis*) and the rhesus macaque (*M. mulatta*). These two species are the widest spread primates next to humans and are categorized as species with Least Concerns by the Red List. Facilitating housing and breeding, macaques live in large multi-male, multi-female groups, mature at the age of app. 3.5 years (great apes: 6–10 years) and have a gestation period of 160–180 days (great apes: 225–270 days), giving birth to one infant per parturition (reviewed in Lindburg, 1991).

Notably, crab-eating macaques have recently been used to model ASDs (Liu et al., 2016; Zhao et al., 2017). Liu and colleagues expressed human MeCP2^32^ in the macaque brain to mimic an ASD resulting from duplications of the *MECP2*-containing genome segment, whereas Zhao and colleagues introduced deletions to *SHANK3*^33^, which are known to cause ASD. Remarkably, both studies found striking differences compared to the corresponding mouse models: while the *MECP2*-overexpression model demonstrated clear autism-like behaviors in primates, but not in mice, the *SHANK3*-deletion model revealed significant differences in the expression pattern of Shank3, and found an impaired neurogenesis in the prefrontal cortex of macaques.

Despite these promising results, macaque models also have some disadvantages: The monkeys are carriers of Herpes B viruses and may transmit this potentially fatal disease to humans. Their long adolescence (3.5 years) hampers breeding, and their longevity (approximately 30 years in captivity) is clearly not supporting studies of senescence. Although highly developed, their social system is not in favor of ASD research as well, since macaques live in large multi-male/female groups, whereas humans live in (extended) family groups. As a result, proactive prosociality, which is believed to be based on shared infant care, is well developed in humans, but not in chimpanzees or macaques (Burkart and van Schaik, 2013).

For these reasons, marmosets have recently garnered interest as model animals for studying brain function (reviewed in Saito, 2015; Tokuno et al., 2015), for a general review on the species, please see (Schiel and Souto, 2017). Also known as zaris, these new world monkeys split from the primate lineage approximately 40 million years ago, 35 million years before humans and chimpanzees separated. Compared to great apes and macaques, marmosets have the advantage of being small, easy to handle, easy to breed and less expensive: with a life span of about 12 years in captivity, marmosets mature more quickly than great apes or macaques, starting reproduction at 18 months of age and giving birth to twins after a gestation period of 140–150 days. Contrary to macaques, marmosets do not represent a natural reservoir of Herpes B viruses.

Though lissencephalic (smooth), their brain shares many features of the human brain, and their cerebral cortex shows the neuronal architecture of all primates (Bendor and Wang, 2005; Burman et al., 2006; Elston et al., 2006; Burman and Rosa, 2009; de la Mothe et al., 2012; Chaplin et al., 2013), from which only humans differ in that neurons of their prefrontal cortex are more spiny and more complex than those of their primate relatives (Elston et al., 2006). Consequently, marmosets have the high cognitive abilities characteristic to all primates: they are not only able to perform true imitation (Bugnyar and Huber, 1997; Voelkl and Huber, 2007), transposition and generalization (Yamazaki et al., 2014) but to also solve string problems (Halsey et al., 2006; Gagne et al., 2012) and to understand physical causality (Yamazaki et al., 2011). Considering the weaknesses of FXS individuals in abstract item reasoning and in addressing new problems, the cognitive abilities of marmosets might help to develop tasks which directly translate results between the two species.

Of particular interest for the design of ASD models is the sociality of marmosets. In a convergent evolution to humans, marmosets developed a high level of group living (reviewed in Graham, 2016). At a state of sophistication not known from other mammals including most primates, marmosets demonstrate altruistic behaviors such as proactive prosociality and third-party reciprocity (Burkart et al., 2007; Burkart and van Schaik, 2013), social learning during infancy (Dell’Mour et al., 2009) as well as adulthood (Caldwell and Whiten, 2004), and behavioral adaptations to social environments (Koski and Burkart, 2015). Similar to humans, marmosets live in extended family groups, pair-bond and care for their offspring in a cooperative manner (Digby and Barreto, 1993; Sousa et al., 2005; Birnie et al., 2013; Yamamoto et al., 2014). Since parental care and in particular maternal warmth are known to influence FXS symptoms in children (Dyer-Friedman et al., 2002; Kuo et al., 2002; Glaser et al., 2003; Greenberg et al., 2012; Robinson et al., 2016; Smith et al., 2016), the latter could be useful to further investigate the role of the social environment for FXS.

Like other primates, marmosets mostly rely on vocalizations (Miller and Wang, 2006; Chen et al., 2009; Miller et al., 2010; Watson and Caldwell, 2010; Bakker et al., 2014; Kato et al., 2014; Agamaite et al., 2015) and visual cues (de Boer et al., 2013; Massen et al., 2016) for their communication, although social grooming (Lazaro-Perea et al., 2004) and scent marking (Epple, 1970; Massen et al., 2016) are present as well. Since ASDs are characterized by gaze avoidance, it is particularly interesting that marmosets are able to use a variety of facial expressions for communication (Kemp and Kaplan, 2013) and to gain information by geometrical gaze following, even from human experimenters (Burkart and Heschl, 2006). Analyzing the face scanning patterns performed by marmosets, Kotani et al. (2017) showed that these primates primarily view the eye region during contact and that this behavior can be employed to evaluate the influence of drugs.

Due to the dense vegetation of their natural habitat, vocalizations are unusual rich in marmosets, even for primates (Epple, 1968; Morrill et al., 2013; Agamaite et al., 2015). Marmoset conversations involve a cooperative vocal control and require the infants to learn when to talk and when to listen (Takahashi et al., 2013, 2016; Choi J. Y. et al., 2015; Chow et al., 2015), thereby paralleling human development. Indeed, recent research revealed that marmoset infants need to transform their babbling and crying into mature vocalizations in order to properly communicate with other group members (Margaret Elowson et al., 1998; Pistorio et al., 2006; Takahashi D. Y. et al., 2015; Ghazanfar and Zhang, 2016) and that this transformation requires social reinforcement from caregivers (Margaret Elowson et al., 1998; Takahashi et al., 2017). Given the ability of marmosets to even learn grammar (Wilson et al., 2013) and the cluttered speech observed in individuals with FXS (Hanson et al., 1986; Belser and Sudhalter, 2001; Roberts et al., 2007; Klusek et al., 2014), the high level of vocal communication in marmosets could be valuable to the development of treatments for FXS and ASD patients.

Though their sociality and their cognitive abilities make marmosets a promising model organism for FXS and ASD research, it is just this very same point, which also bears the disadvantages. The high level of communication and understanding of social interactions increases the risk that relations between researchers and experimental animals arise, which may impact on experiments (cp. Herzog, 2002), for example, if individual animals dislike or prefer being handled or cared for by a certain person and subsequent stress levels influence the outcome of an experiment. Since the n-numbers used in primate research are at the minimum, such effects might easily turn out “significant”.

Another important concern is morality. Although all mammals are able to feel pain, stress and fear in a similar way, no matter whether they are rodents, primates or of any other clade, the high cognitive capabilities and social skills of primates have raised particular ethical concerns regarding their use in biomedical research (cp. the reviews of Coors et al., 2010; Phillips et al., 2014; Zhou, 2014; Bailey and Taylor, 2016; Arnason, 2017). As a consequence, many countries have implemented policies and regulations to ensure the physiological as well as psychological well-being of all research animals (Pereira et al., 2004; Luy, 2007; Hansen et al., 2017), and the standards are particularly high for primates (see Tardif et al., 2013; Weiss and Hampshire, 2015). However, the ethical viewpoints often differ, not only within a society (cp. Buckley et al., 2011; Phillips et al., 2014; Arnason, 2017 vs. Bailey and Taylor, 2016), but also among societies (cp. Cyranoski, 2004; Zhang X. L. et al., 2014), causing the hurdles for the approval of a project to vary significantly between countries and sometimes even within countries. The same project might thus be approved within a few weeks in one country, within a few months in another, and not in years in the next.

These circumstances raise several questions:

Is it ethically justified to approve therapies developed in countries with lower animal welfare standards in countries with higher standards, considering that such approvals would torpedo all animal welfare efforts? Is it ethically justified to deny patients a therapy developed in a country, which had lower animal care standards? Is it ethically justified to have wealthy people traveling to countries offering corresponding therapies, while others cannot afford to do so?

Moreover: can cognitive abilities indeed be a measure to define welfare standards and requirements? Where is the red line? These two questions are intimately connected, since the use of cognitive capabilities as an argument for increased standards in turn causes diminished abilities to result in the opposite. Since everything goes the way of least constrains, this is the point where the seemingly high moral standards cause ethics and science to start loosing ground: is it ethically and scientifically justified to use thousands of mice in preclinical research, to enrol hundreds of patients in clinical studies, to put patients at risk, albeit the risk might be marginal, and to cause hopes and disappointments in their families, just to learn how hard it is to find out how to keep the Concorde flying from studying a biplane?

## Author Contributions

RD wrote the manuscript.

## Conflict of Interest Statement

The author declares that the research was conducted in the absence of any commercial or financial relationships that could be construed as a potential conflict of interest.

## Footnotes

^1^intelligence quotient scores

^2^compare

^3^Electroencephalography

^4^untranslated region

^5^5′-C-phosphate-G-3′

^6^single-stranded DNA

^7^Amyloid-Precursor-Protein

^8^In 2006, an inducible Fmr1^−/y^ mouse model was generated to address aspects related to developmental or regional expression patterns, but the animals have only been used in a very few studies thus far, possibly due to limitations in the availability of appropriate Cre-lines. For more information, please refer to Table 2.

^9^metabotropic glutamate receptors 1 and 5, = group 1 mGluRs

^10^α-amino-3-hydroxy-5-methyl-4-isoxazolepropionic acid receptor

^11^extracellular signal-regulated kinase

^12^mammalian target of rapamycin

^13^Phosphoinositid-3-Kinase

^14^PI3K Enhancer

^15^Glycogen synthase kinase 3

^16^long term potentiation

^17^long term depression

^18^N-methyl-D-aspartate receptor

^19^Cornu Ammonis region 1

^20^2-Methyl-6-(phenylethynyl) pyridine

^21^sodium-activated potassium channel Slack

^22^large-conductance calcium-activated potassium channels

^23^gamma-aminobutyric acid

^24^hyperpolarization-activated cyclic nucleotide-gated cation channel

^25^high endothelial venule protein, also known as SPARC like protein

^26^secreted protein, acidic and rich in cysteine

^27^postnatal day 7

^28^functional magnetic resonance imaging

^29^knock-out

^30^zinc-finger nucleases

^31^transcription activator-like effector nucleases

^32^methyl CpG binding protein 2

^33^SH3 and multiple ankyrin repeat domains 3, alias: proline-rich protein 2
